# PortaDrop: A portable digital microfluidic platform providing versatile opportunities for Lab-On-A-Chip applications

**DOI:** 10.1371/journal.pone.0238581

**Published:** 2020-09-03

**Authors:** Tom Kremers, Sarah Thelen, Nils Bosbach, Uwe Schnakenberg

**Affiliations:** Chair of Micro- and Nanosystems and Institute of Materials in Electrical Engineering 1, RWTH Aachen University, Aachen, Germany; University of Glasgow, UNITED KINGDOM

## Abstract

Electrowetting-on-dielectric is a decent technique to manipulate discrete volumes of liquid in form of droplets. In the last decade, electrowetting-on-dielectric systems, also called digital microfluidic systems, became more frequently used for a variety of applications because of their high flexibility and reconfigurability. Thus, one design can be adapted to different assays by only reprogramming. However, this flexibility can only be useful if the entire system is portable and easy to use. This paper presents the development of a portable, stand-alone digital microfluidic system based on a Linux-based operating system running on a Raspberry Pi, which is unique. We present “PortaDrop” exhibiting the following key features: (1) an “all-in-one box” approach, (2) a user-friendly, self-explaining graphical user interface and easy handling, (3) the ability of integrated electrochemical measurements, (4) the ease to implement additional lab equipment via Universal Serial Bus and the General Purpose Interface Bus as well as (5) a standardized experiment documentation. We propose that PortaDrop can be used to carry out experiments in different applications, where small sample volumes in the nanoliter to picoliter range need to be handled an analyzed automatically. As a first application, we present a protocol, where a droplet is consequently exchanged by droplets of another medium using passive dispensing. The exchange is monitored by electrical impedance spectroscopy. It is the first time, the media exchange caused by passive dispensing is characterized by electrochemical impedance spectroscopy. Summarizing, PortaDrop allows easy combination of fluid handling by means of electrowetting and additional sensing.

## Introduction

**Fig 1:**
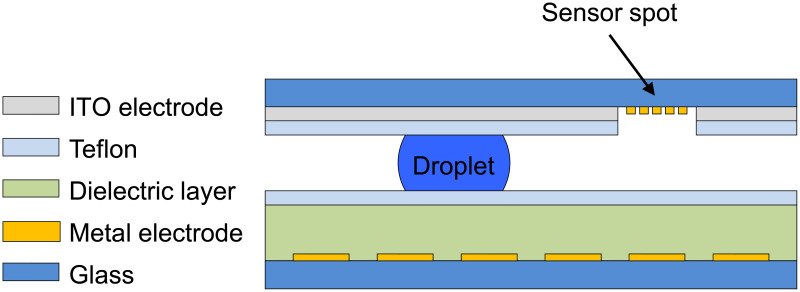
DMF device cross section. Schematic of a commonly used DMF device in cross sectional view using bottom and top plates for droplet manipulation by EWOD. Drawing not to scale.

In digital microfluidics (DMF), discrete nL-droplets of reagents are controlled by applying a series of electrical potentials to an array of electrodes coated with a hydrophobic insulator. The underlying principle is called electrowetting (EW) or electrowetting-on-dielectrics (EWOD). Drop creation, transport, merging, mixing and dispensing of droplets have been demonstrated [1]. Excellent reviews on EWOD and DMF discussing different application areas have been published over the recent years [2–13]. A commonly used DMF device, schematically shown in Fig 1, consists of two parallel-adjusted glass slides with a slit for droplet transport, called closed configuration. The bottom plate comprises individually addressable, square-shaped metal path electrodes, a dielectric layer and a thin hydrophobic layer, typically made of spin-coated Teflon AF films, which cover the surface. The top glass slide is covered with a thin, transparent, non-structured indium-tin-oxide (ITO)-electrode serving as ground electrode. The layer is passivated with a thin Teflon layer, too. In addition, the ITO and Teflon layer can be patterned to form locally hydrophilic spots. This allows the integration of sensor elements, where the droplet tears-off while being moved across and building a column of media underneath the sensor element, which is termed virtual microwell. The underlying mechanism is called passive dispensing [14]. Spacer structures separate both slides and guarantee parallel plates. In comparison, the open DMF configuration operates without the top plate. Here, only the electrodes at the bottom plate are addressed for initiating droplet movements across the surface [3].

In the closed configuration, droplet movement is initiated by application of typically AC potentials between a path electrode and the ground electrode driving the droplet to the charged electrode. The charge imbalance at the liquid-dielectric interface results in an electrostatic (Coulomb) force that causes changes in surface tension between solid and liquid phase and in the corresponding contact angle [15–22]. Because the liquid is not in direct contact with the path and ground electrodes, electrolysis or analyte redox reactions are avoided, enhancing the device stability. Contact angle changes do not depend on the applied frequency [23] but on the voltage squared according to Young-Lippmann equation [1, 17, 24–32]. Droplet volumes down to picoliter range [21, 33] and non-aqueous solvents and solutions [23] have already been manipulated successfully. DMF platforms benefit from the flexible reconfigurability and reprogrammability of the path electrodes.

For operation, commonly used lab bench-based DMF systems need bulky peripheral equipment such as power supply, function generator, multiplexer, voltage amplifier and high-voltage pulse generator. To address point-of-use systems, some attempts have already been made for the development of portable DMF systems. Gong et al. published the first portable DMF device by using a double-sided printed circuit board (PCB) with batteries, droplet and sensing circuits, a microprocessor and an infrared user interface in combination with land grid array sockets to mount the DMF chip [34, 35]. Using a Palm Vx PDA (Personal Digital Assistant), the droplet movement and position were controlled wirelessly via a feedback control.

Sista et al. published a DMF cartridge based on a 384-well plate format with 12 sample and 8 reagents reservoirs [36]. The format of the cartridge is compatible with high-throughput robotic systems. Heating bars of aluminum in combination with PID-controlled thermistors are connected on the cartridge and enable controlled and localized heating. The cartridge is placed in a control instrument to the electronic controller by spring-loaded connector pins. The controller consists of a microprocessor and a switching circuitry to control up to 64 actuation electrodes by a custom written software. Unfortunately, no specifications about hard- and software were presented. Using the control instrument, chemiluminescence, using a photon counting multiplier tube, as well as fluorescence, using an integrated fluorimeter that comprises a light emitting diode and a photodiode, were detected.

The portable DropBot DMF system was introduced by the Wheeler Microfluidics Laboratory at the University of Toronto, Canada, where the DMF chip is connected via pogo-pins to a PCB board interface and then to an Arduino-based control box with up to eight 40-channel high-voltage driver boards [37]. An Arduino Mega 2560 microcontroller generates a square-wave signal, which is amplified by the high-voltage amplifier and transferred via a control board to the actuation pads. With DropBot, the drop position and instantaneous its velocity can be monitored by measuring the impedance between path and top electrode instead of using high-speed cameras. Furthermore, DropBot allows automated, real-time tuning of applied potentials to maintain a constant driving force F_DR_ = 0.5 L C V^2^ with L electrode width, C capacity per unit area and V applied voltage, respectively, regardless of device-specific properties, e.g., differences in thickness and dielectric constant of the insulator and drop composition. To carry out electrochemical measurements, the open-source potentiostat DStat is used [38]. DStat enables amperometric and voltammetric measurements, is connected via Universal Serial Bus (USB) and is running with a custom-made software on a computer. The system can operate with DropBot via a plug-in in the DropBot software “μDrop” using the high-performance asynchronous messaging library ZeroMQ [37, 38]. Therefore, DropBot pauses the droplet actuation, sends a request to the DStat and waits until the electrochemical measurement is performed. Recently, DropBot and DStat were used in a “plug-n-play” DMF (PnP-DMF) system, were the commonly used top glass slide was exchanged by a poly(methyl methacrylate) (PMMA) backing and a glued ITO-coated poly(ethylene terephthalate) (PET) film coated with hydrophobic Flouropel. The PMMA slide was laser cut to seamlessly couple prefabricated sensors by inserting the sensor chips into the slit [39].

The OpenDrop approach was developed for manipulation of droplets in an open device configuration [40]. The glass plate with the 64 actuation electrodes, the control unit with the step-up DC-DC power converter (up to 260 V), the high-voltage switches (surface-mount transistors) and regulator are connected to a two-layer PCB board and controlled by a tablet or smartphone with touch screen capability. The visual interface allows the setting of drop pathways and merging as well as splitting by simple finger dragging. The latest version, OpenDrop3, needs only 5 V power supply via an USB connector. It can address 14x8 electrodes with voltages up to 300 V via a 32bit microprocessor, has electronic settings of voltage level, frequency and AC/DC selection as well as electronic reading of the actual voltage level [41]. Compared to the first version, the ITO-coated top glass plate can be connected to allow droplet manipulation in a closed DMF configuration. In comparison, OpenDrop consists only of a small cartridge connected to a tablet, whereas DropBot needs the described peripheral space-filling equipment.

The cartridge-based Sandia Digital Microfluidic Hub introduces a capillary interface enabling highly repeatable transfer of liquid between the DMF device and external fluidic modules, like external pumps, valves, and reservoirs for precise reagent dispensing [42]. The Teflon-coated capillaries are adjusted between the two glass plates. Droplet dispensing is sensed by sample conductivity measurements. Above a threshold, the software automatically triggers droplet actuation. Droplets are actuated by applying optimized AC voltage pulse (typically 50–105 V_rms_ at 15 kHz) to the electrodes. A computer-controlled electronic interface either was implemented to activate pads by manual keystrokes or by predetermined script sequences. Peripheral devices were not presented by Kim et al., because they focused on the new functionality of the “fluid distribution hub”.

Yafia et al. published a low-cost portable DMF platform in combination with a smartphone. Here, two small lithium ion batteries serve as the power supply. A boost DC-DC converter amplifies the input voltage of 8 V to 800 V output voltage, which is controlled by either changing the pulse switching frequency or the duty cycle [43]. An Arduino-Micro controls the high-voltage switching circuitry for addressing the pads and is used for the wireless communication with a smartphone via a Bluetooth module [43]. Besides sending serial commands to the microcontroller, the smartphone is also used as a detection and imaging analysis station for calorimetric measurements.

A similar approach of using a smartphone for controlling droplet movements and detecting chemiluminescence was published by Zeng et al. [44]. They developed a low-cost, portable DMF analysis platform in open configuration with three power supplies and an ICL8038 signal generator module, which generates a voltage of 12 V_rms_. This square wave is amplified to 45 V_rms_ by the high-voltage amplifier PA443 from Apex Microtechnology, USA, and then coupled to a relay array. In the original version, only 10 electrode pads are addressed but the develop board can control up to 46 channels at the same time. Only 45 V_rms_ are needed as the droplet driving voltage because the bottom glass plate is coated with 1 μm thick cyanoethyl pullulan as a high-k dielectric layer [45]. The developed control board is an open-source board named I.O.I.O.-OTG and provides a high-level Java API that manipulates I/O ports by a smartphone.

Recently, Hoang et al. published a portable DMF platform consisting of four main components: a DMF chip, an Arduino-Nano-based control board, a user interface and a heating unit for loop-mediated isothermal amplification (LAMP) reactions [46]. The control board also incorporates a high-voltage DC-DC power converter, which is capable of stepping up to 275 V from a 12 V input. A 64-channel and 300 V-rating serial-to-parallel shift register HV507 (Microchip Technology Inc.) is used as an interface between the 5 V-compatible microcontroller and the high-voltage power line [46]. The HV507 allows for the control of up to 64 electrodes. Because the HV507 is not compatible with AC signals, a complicated solution is presented to address the electrodes individually [46]. A similar approach was published by Wan et al. [47]. Unfortunately, they did not present detailed information about the used hard- and software.

In the portable DMF platform developed by Coudron et al. an 18F45K22 microcontroller contains an 8-bit sine wave lookup table and output to an R-2R resistor ladder to produce a 1 kHz analog sine wave. After amplification, up to 225 V_rms_ have been applied to 48 driving electrodes individually by a digital potentiometric control and use of high voltage MOSFET switches [48].

Sensors integrated in DMF systems are rather rare. With regard to electrochemical sensors, De Campos et al. recently published a flexible “plug-n-play” solution for embedding commercially available electrochemical sensors and presented amperometric detection of glucose, β-ketone and lactate [39]. Amperometric detection was utilized to monitor the oxidation of 3,3′,5,5′-tetramethylbenzidine (TMB) by a horseradish peroxidase (HRP) [49]. TMB is commonly used in an enzyme-linked immunosorbent assay (ELISA) and is used for, e.g., end-point-detection of the final preparation state of a protein stack by photometric measurements [50, 51]. Farzbod et al. demonstrated the integration of a potassium selective sensor [52]. They integrate an Ag/AgCl on-chip pseudo-reference electrode into the DMF device and use voltammetry achieving a sensor response close to Nernstian behavior. Yu et al. monitor the dopamine uptake in neurons by cyclic voltammetry (CV) integrated in DMF [53, 54]. Dryden et al. use integrated electrodes for linear sweep voltammetry (LSV) measurements of different acetaminophen concentrations generated by repeated dilution using the EWOD fluid handling capabilities bearing a limit of detection of 76 μM [55]. Nanostructured microelectrodes providing a high surface area were integrated by Rackus et al. into DMF leading to a high sensitivity in the application of an electrochemical assay for rubella virus immunity [56]. They use differential pulse voltammetry (DPV) for analysis of the samples. Approaches to monitor the cell density and thus the cell proliferation by impedance sensing or to measure the electrical properties of the droplet through the insulating layer have also been shown [57, 58]. The combination of an open EWOD system in form of a T-junction mixer and external electrochemical sensors has been was described as well [59]. A different approach, where an ITO working electrode is integrated in the top chip, was presented by Shamsi et al. [60]. They use electrochemiluminescence to detect single nucleotide differences in microRNA allowing differentiation between MDA-MB-231 and MCF-7 breast cancer cells.

To summarize, portable DMF platforms comprise of five main components: a DMF chip in open or closed configuration, a power supply, a signal generator when AC signals are preferred, a high-voltage generator for droplet actuation and a microcontroller in combination with a switching board to address the electrodes individually. However, the presented approaches all deliver these needs but are lacking the capability to easily extend the setup with analysis devices used for different applications.

Here, we present PortaDrop, a portable DMF platform. PortaDrop operates with Raspbian, a Debian-based Linux distribution especially developed for the use with a Raspberry Pi. The benefit of the Raspberry Pi is the ease of adding external measurement systems via the General Purpose Interface Bus (GPIB). Therefore, an USB-to-GPIB adapter can be connected to the Raspberry Pi. The control of the entire system is possible via a graphical user interface (GUI) which is optimized for a 7” liquid crystal display (LCD) touchscreen.

The presented system can operate stand-alone requiring only a 5 V USB power supply. PortaDrop provides all electronic components for controlling droplets with up to 118 path electrodes in open and closed configuration, including a powerful boost converter and a voltage rectifier. Electrochemical measurements like LSV, CV, Square Wave Voltammetry (SWV), DPV, Normal Pulse Voltammetry (NPV), Chronoamperometry (CA), Open Circuit Potentiometry (OCP), MultiStep Amperometry (MA) and electrochemical impedance spectroscopy (EIS) can be carried out using the integrated EMStat Pico. Here, in the first version of PortaDrop, we focus on the implementation of EIS.

The paper is structured as follows: First, the setup and the hardware of PortaDrop is presented followed by a description of the software and the GUI frontend. Then, the performance of the internal hardware in interaction with the software is described and the achievable droplet velocity is investigated. For proof-of-concept, a media exchange via passive dispensing is monitored by EIS for the first time.

## Experimental setup

### Hardware

**Fig 2:**
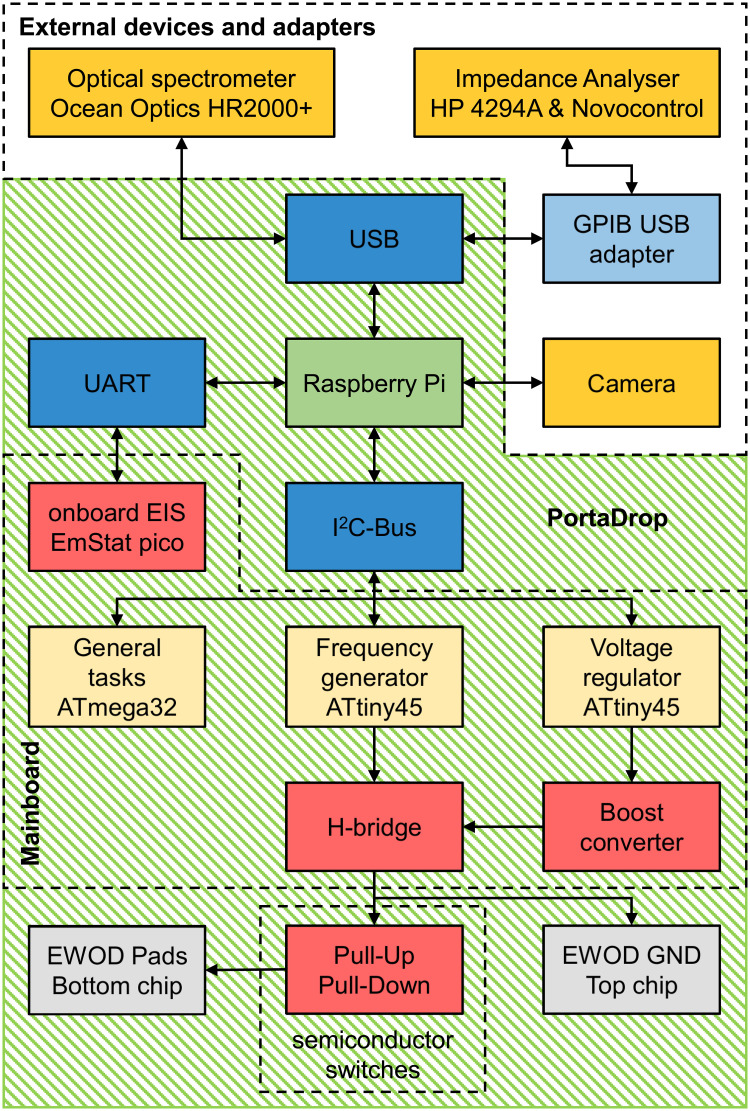
Schematic block diagram of PortaDrop. The hardware implemented on the mainboard and external devices and adapters are bordered in dashed lines. The green area indicates the main box of PortaDrop offering EWOD operation and integrated electrochemical measurements.

The hardware of PortaDrop consists of two different circuit boards: first, a mainboard for the management of all functionalities of the system, which is communicating with the Raspberry Pi 3B+ (Raspberry Pi, purchased at Reichelt Elektronik GmbH & Co. KG, Sande, Germany) via Inter-Integrated Circuit (I^2^C)-Bus, and second, a board including semiconductor switches operating as pull-down and pull-up networks to activate and deactivate the electrowetting path electrodes on the bottom chip. One board is capable to control up to 59 path electrodes. Multiples of the second board can be connected to the mainboard. The entire hardware of PortaDrop is supplied by only one 5 V USB switching power supply and the power is distributed to all units of the system. A schematic of the system is depicted in Fig 2. The green area represents the main box of PortaDrop, which is completely portable and provides all functionalities needed for EWOD operation as well as integrated electrochemical measurements using the EmStat pico.

**Fig 3:**
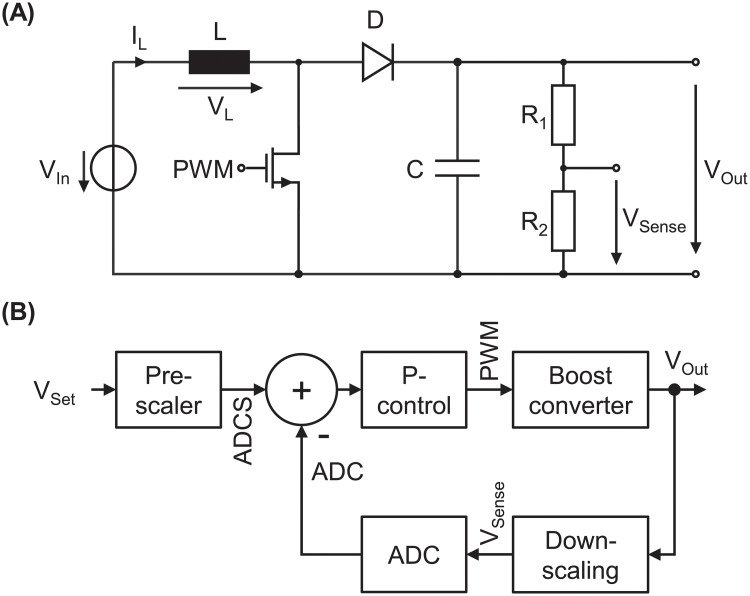
Boost converter. (A) Schematic of the developed boost converter (B) Closed control loop of the boost converter to allow automated voltage level regulation independent of the load.

In the following, the hardware is presented in more detail. As already mentioned, relatively high and preferred alternating voltages are required for droplet actuation. To achieve this, the mainboard consists of two circuit blocks: (1) a controlled boost-converter to generate high DC voltages up to 400 V and (2) an H-bridge to rectify the output voltage of the boost converter. As indicated in Fig 2, the Raspberry Pi communicates via I^2^C with two ATtiny45 microcontrollers: a frequency generator and a voltage regulator. This enables the generation of the AC high-voltage independent of the Raspberry Pi, which only sends the set point for frequency and voltage. Both microcontrollers take care of their specific tasks, individually. In addition, both microcontrollers measure the actual frequency or voltage, respectively, and make the value accessible via I^2^C. A schematic of the boost converter implemented on the mainboard and the closed control loop to adjust the voltage are depicted in Fig 3.

The boost converter consists of a power N-Channel MOSFET (FCP20N60, 600V, 20A, TO-220, purchased at Farnell GmbH, Aschheim, Germany) and standard circuit elements (L = 10.38 mH (DC-resistance 34.7 Ω), D = 1N4007, C = 10 μF (450 V)). The architecture of the boost converter provides the advantage that the output voltage is a function of the duty cycle of the pulse width modulation (PWM) signal at the gate of the N-Channel MOSFET switching the transistor on and off [61]. The microcontroller receives the voltage set point and stores the value in a register being accessible via I^2^C. A pre-scaling factor considers the analog-to-digital converter (ADC) resolution of 10 bits, the down-scaling factor due to the voltage divider (factor 101) and the reference voltage of the ADC (5V). A simple proportional controller (P-control) adjusts the duty cycle of the PWM signal leading to a change in output voltage V_Out_ of the boost converter. V_Out_ is scaled down to V_Sense_ by the voltage divider made of R_1_ (30 MΩ) and R_2_ (300 kΩ) to protect the microcontroller from damage due to overvoltage. The measured voltage V_Sense_ is AD converted and compared to the stored set point ADCS. Then again, the duty cycle is adjusted and the boost converter changes the output voltage. It is worthwhile to note that this kind of control is able to react on changes in load like enabled and disabled electrowetting path electrodes.

**Fig 4:**
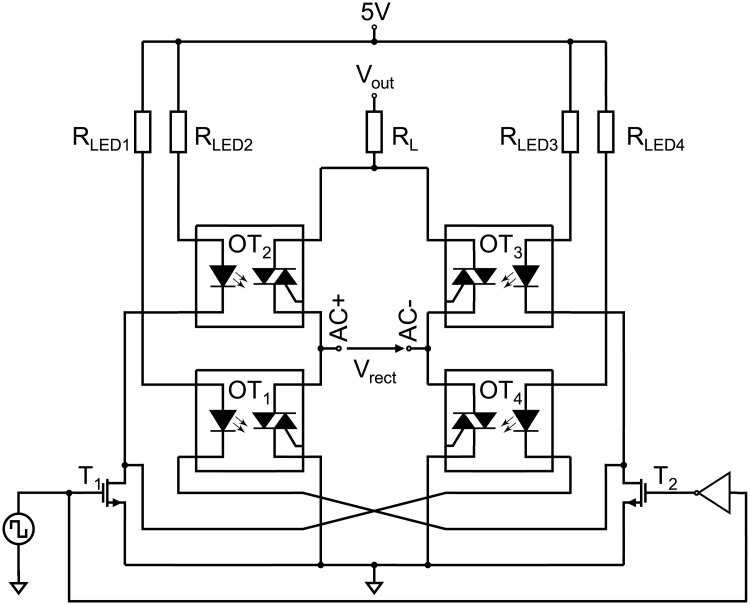
H-bridge. H-bridge circuit to generate a bipolar high-voltage rectangle signal of the amplitude V_out_. The optotriacs OT_2_ and OT_4_ or OT_1_ and OT_3_ are alternately switched to conducting state by a TTL rectangle signal provided by the microcontroller ATtiny45.

To supply an alternating voltage to the path electrodes, the DC output voltage of the boost converter is chopped by an H-bridge circuit shown in Fig 4.

The H-bridge consists of four semiconductor switches. Optotriacs (MOC 3041, Motorola, purchased at Reichelt Elektronik GmbH & Co. KG, Sande, Germany) are used because of their high voltage stability up to 400 V and the galvanic decoupling between the high-voltage terminals and the light emitting diode (LED) side, where transistor-transistor-logic (TTL) levels are used to control the signals. The H-bridge is supplied with 5 V at the low-voltage part of the circuit and the output voltage V_out_ of the boost converter at the high-voltage part, respectively. Transistor T_1_ controls the optotriacs OT_2_ and OT_4_, while T_2_ controls OT_1_ and OT_3_, respectively, by a signal of opposite phase indicated by the inverter at the gate of T_2_. The rectified voltage V_rect_ equals V_out_ when T_1_ is biased and thus T_2_ is off, while V_rect_ is equal to the negative output voltage of the boost converter when the logic level changes from *HIGH* to *LOW* and vice versa. It is worthwhile to note that the gate signal of T_1_ is not entirely inverted to T_2_. In case of using a CMOS inverter, the condition that both transistors and thus all optotriacs being (partly) conducting arises, which shortens V_out_ and *GND*. Instead of an inverter, we utilize two individual microcontroller pins, where the pins are toggled with a delay of 375 ns. The delayed switching behavior is depicted in S1 Fig in S1 File. Although, the optotriacs exhibit high-voltage stability, the maximum slew rate of the device is limited, and device failure occurs above 1000 V/μs. To prevent device failure, the limiter resistance R_L_ (44 Ω) is introduced adding an artificial delay to the recharging of the output terminals AC+ and AC-. AC- is here referred to the potential of the counter electrode in electrowetting operation, while AC+ is the potential applied to the path electrodes activating them.

**Fig 5:**
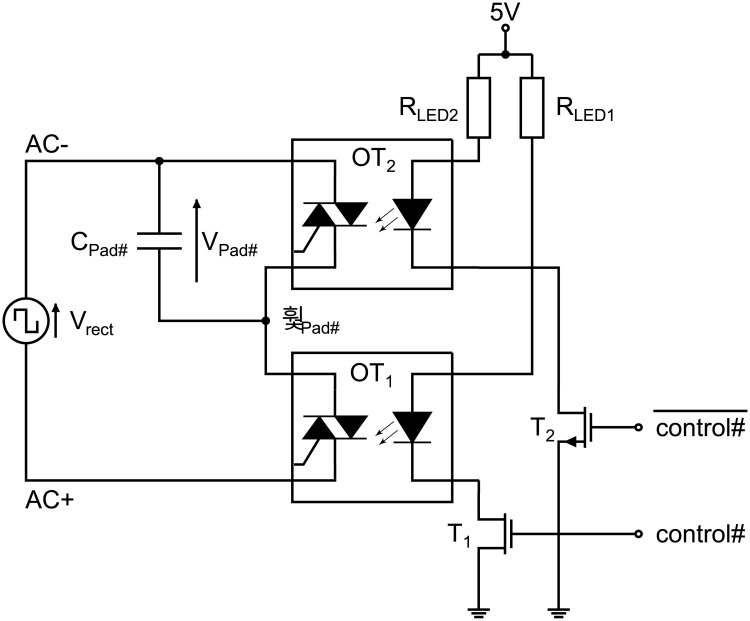
Pull-down and pull-up network for EWOD pad control of one path electrode. The capacity C_Pad#_ represents the path electrode and the counter electrode. By switching on OT_2_, the capacity is shortened and the corresponding path electrode deactivated. The path electrode is activated by making OT_1_ conductive instead of OT_2_ to apply the bipolar high-voltage signal to the Pad. Droplets overlapping the electric field lines will then be attracted, if a sufficiently high potential is applied.

The second board contains the semiconductor-based pull-down and pull-up networks to activate and deactivate the electrowetting pads. This PCB board is connected using a 4-pole connector cable (Sensor/Actuator cable—SAC-4P-M12MS /0,3-186/M12FS–1509526, Phoenix Contact, purchased at Conrad Electronic SE, Hirschau, Germany) to the mainboard, which supplies the switching units with 5 V bias and the potentials AC+ and AC- for all pads. Multiple boards can be stacked to increase the number of controllable EWOD pads. Here in the presented version of PortaDrop, only one board is connected. The schematic of a pull-down and pull-up network for a single path electrode is depicted in Fig 5.

Each path electrode and the counter electrode represent a capacity C_Pad#_. In case of a deactivated pad, the logic level at the gate of T_1_ (control#) is *LOW* and the inverted signal indicated with a dash is *HIGH*. Both terminals of C_Pad#_ are connected to AC- by OT_2_. No potential difference across the pad capacity occurs. Inverting control#, the transistors toggle their state and the path electrode indicated with *ϕ*_Pad#_ is connected to AC+. Both control signals per single pad switching unit are provided by an ATmega32 microcontroller, which receives the number # of the pad that should be activated or deactivated, respectively. Each microcontroller of in total five on one semiconductor switches board is in charge of 12 pads (note: one of the five microcontrollers is only connected to 11 pads due to wiring restrictions), allowing the control of the entire board. Communication between the microcontrollers and the Raspberry Pi is carried out through a parallel protocol, which is implemented in software and uses the General-Purpose Input Output (GPIO) pins of the Raspberry Pi and the microcontroller. The pins are connected via a single ribbon cable. Again, two microcontroller pins are used for control# and inverted control# to avoid a short between AC- and AC+ changing the state with a time delay (compare S1 Fig in S1 File).

In addition to the voltage generation and the switching circuits for the EWOD pads (described above), the mainboard provides jacks for operation with an external high-voltage instead of the internal boost converter, four BNC connectors to use external devices for electrochemical measurements and SMA connectors to connect electrodes for these measurements, e.g. on the top chip. For electrochemical measurements without additional external devices, the integrated commercially available potentiostat EMStat Pico (Palmsens BV, Houten, The Netherlands) is mounted on the mainboard. For communication with the module, the internal Universal Asynchronous Receiver Transmitter (UART) interface of the Raspberry Pi is used. Furthermore, peripherical circuit blocks are implemented on the mainboard: commercially available step-up converter generating 12 V for the boost converter input, level shifter to mediate between 3.3 V and 5 V TTL voltage levels (see S2 Fig in S1 File), shift registers to control status LEDs, a Raspberry Pi Real Time Clock Module (Raspberry Pi, purchased at Reichelt Elektronik GmbH & Co. KG, Sande, Germany), humidity and temperature sensors (DHT11, JOY-IT, purchased at Reichelt Elektronik GmbH & Co. KG, Sande, Germany), an ATmega32 microcontroller taking care of general tasks individually receiving instructions from the Raspberry Pi via I^2^C and mechanical relays (Finder 36.11.9.012.4011, purchased at Farnell GmbH, Aschheim, Germany). The relays are biased with 12 V and ensure galvanic decoupling when needed. A sketch of all relay functions is presented in S3 Fig in S1 File. Briefly, the relays on the mainboard can be divided in three groups: (1) switching-off all paths for high-voltage supply and rectifier, (2) changing the routing between EWOD operation and EIS measurements and (3) switching between internal and external EIS devices in 2-, 3- or 4-probe setup. Important to note is the ability of connecting the boost converter output to a resistor, which is not only important to discharge the energy stored in the output capacity, but also allows the voltage regulator to quickly decrease the output voltage. In comparison, discharging only by power consumption of the load is much slower and thus the voltage set point would not be reached in time.

### Software

The main software of PortaDrop runs on the Raspberry Pi (running the Debian-based operating system Raspbian) and is written in the high-level language C++. The entire source code and an installation readme is available in S1 Source code and on GitHub (https://github.com/ewodac/PortaDrop). The software exhibits a GUI providing complete control about the system. The GUI was created using the open-source cross-platform widget toolkit GTK in addition with the User Interface Designer Glade. The main window (Overview → General) of the GUI is presented in S4 Fig in S1 File. In addition, screenshots and detailed information of the different tabs and options are given in S5 to S20 Figs in S1 File and are described in S1 Appendix in S1 File.

The GUI is optimized for the use on the internal 7” touchscreen (Raspberry Pi, purchased at Reichelt Elektronik GmbH & Co. KG, Sande, Germany), but also works well with external monitors connected via High Definition Multimedia Interface (HDMI) to PortaDrop. The software offers a simple way to plan, execute and control droplet transport and additional measurements, e.g. EIS. All data obtained during an experiment (video, logfile, measurements) can be stored, reviewed and afterwards copied to other computers or USB devices for post-processing. Due to the use of a Linux-based system, the platform can be easily integrated in an ethernet or wireless network to share data with other computers in the network or control the system from remote. The chosen data formats are commonly known and platform independent (*.mp4, *.txt, *.csv). Recipes can be planed and stored in *.xml format and for sure composed assisted by the GUI without any knowledge about the file structure of *.xml. An example of a recipe is given in the supporting information and comments are inserted for brief explanation (S2 Appendix in S1 File).

For the control of external measurement devices, the GPIB standard can be used. The National Instruments GPIB-to-USB adapter NI GPIB-USB-HS can be accessed using the Linux-GPIB library [62]. The library offers a C++ interface to send GPIB commands to connected devices. To distinguish different devices on the same bus, the GPIB address is used. Since different devices support different GPIB commands, each device, which should be accessed via GPIB by PortaDrop, needs to be implemented by a software engineer once. The first version of PortaDrop supports two GPIB devices: Novocontrol Technologies Alpha-A High Performance Frequency Analyzer (Novocontrol Technologies GmbH & Co. KG, Montabaur, Germany) and Hewlett Packard 4294A Precision Impedance Analyzer (Palo Alto, CA, USA) with fixed slave addresses. Additional devices can easily be added in form of a software update. In addition to the external devices, the potentiostat EmStat Pico is mounted on the mainboard and connected to PortaDrop via the UART interface of the Raspberry Pi using the Serial Library of the WiringPi project for communication [63].

The optical USB spectrometer OceanOptics HR 2000+ (Ocean Insight, FL; USA) communicates via USB and can be accessed by the C++ program by the library SeaBreeze [64]. PortaDrop supports the configuration of the measurement and capturing of a spectrum. S21 Fig in S1 File shows an exemplary measurement of four different LEDs taken by the implemented spectrometer. Optical spectra measurements can be useful in future, e.g. for localized surface plasmon resonance (LSPR) measurements within the EWOD systems. The use of DMF systems in combination with LSPR has been shown by Malic et al. [65–68]. This feature is not further used in frame of this work but shows the possibilities of a simple extension of PortaDrop.

PortaDrop is controlled through tasks. Tasks can be organized using recipes which are collections of tasks or other recipes that should be executed in sequence. The current version implements the following tasks: (i) ‘Pad Task’, which activates specified path electrodes for a given time, (ii) ‘Frequency Task’, which sets the frequency of the rectangular signal generator, (iii) ‘Voltage Task’, which adjusts the set point of the boost converter controller, (iv) ‘Spectrometer Task’, which captures an optical spectrum using the OceanOptics spectrometer, (v) ‘Impedance Task’, which captures an impedance spectrum using an external GPIB impedance analyzer or the internal EmStat Pico, and (vi) ‘Delay Task’, which delays the execution of the next task in a recipe for a given time.

### All-in-one box

**Fig 6:**
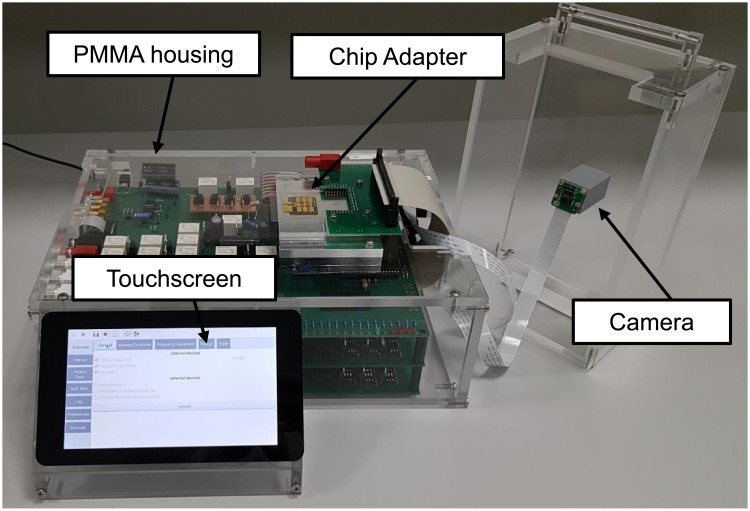
Photo of the assembled PortaDrop in operation. The camera included in the lid can be placed on top of the box and provides a video of the experiment.

All parts are assembled in a custom-made PMMA case; the circuit boards are screwed to the bottom plate of the box and stacked. For all connectors to the Raspberry Pi (4x USB, 1x LAN, 1x HDMI) and for the connectors to the mainboard (4x BNC, 4x SMA, 1x USB power supply, jacks for external high-voltage supply), slits and drill holes have been arranged corresponding to their positions on the board. The semiconductor switching board provides a 60-lead ribbon cable with 59 controllable lines to connect the EWOD path electrodes on the bottom chip (note that line 60 is always set to the counter potential AC-). The 7” touchscreen is placed at the front of the PMMA box and tilted by 45° to allow good control of the operator in front of the PortaDrop system. The assembled box is shown in Fig 6. The dimensions of the box without lid and touchscreen are 34.8 cm x 25.4 cm with a height of 11.4 cm. The touchscreen panel is 19.5 cm in width and protrudes 8.7 cm to the front. The total weight of PortaDrop is only 5.7 kg.

On top of the housing, a spot for the EWOD chips in open or closed configuration is defined. Since our aim is to provide a versatile system, the holder is exchangeable, perfectly fitting to the need of every specific application. A close-up of the holder is depicted in S22 Fig in S1 File. In general, the PortaDrop box provides the complete functionality, the experimental setup can be customized on top of the box as long as certain constraints are fulfilled, e.g. the use of the arranged threads in the top plate to tighten the adapter or the use of the 60-lead ribbon cable. On top of the box, a lid is fixed to secure the setup from contamination and to avoid contact of the operator with the high-voltage. For every application, the lid can be customized as well. Here, as an example, only a holder for the 8 megapixel (MP) Raspberry Pi camera (Raspberry Pi, purchased at Reichelt Elektronik GmbH & Co. KG, Sande, Germany) is placed over the EWOD chips, which is connected via the Camera Serial Interface (CSI) to the camera port of the Raspberry Pi to record the experiments. For different applications, it is possible to add different components, e.g. a fiber to connect the implemented USB spectrometer. The bottom chip holder used here is made of aluminum. A PCB with pogo pins connects the contact pads at the bottom chip to the 60-lead ribbon cable. For fixation of the top chip, a 3D-printed fixture is designed, which can be easily slided into the holder of the bottom chip aligning both. A photo of the top chip adapter and the electrical connections for EWOD operation and EIS measurements is shown in S23 Fig in S1 File and an assembly together with the bottom chip in S24 Fig in S1 File. S1 Video visualizes the loading of two reservoirs using the fixture. Of course, it is still possible to define the spacer between bottom and top chip with e.g. Parafilm or double-sided tape. However, this requires more handling experience [39, 69–84].

## Results

First, the implemented hardware in combination with the software is being tested in terms of performance. Therefore, a predefined voltage profile is executed, and the response of the boost converter is measured. In addition, the output of the rectifier in combination with the semiconductor switching circuit is investigated. The fluid handling capabilities is shown by investigating the dispensing accuracy and the maximum achievable droplet velocity. Finally, an experiment is presented, in which a droplet of phosphate-buffered saline (PBS) is delivered to the EIS sensor in the top plate by EWOD and consequently exchanged by multiple exchange droplets of deionized water using passive dispensing.

**Fig 7:**
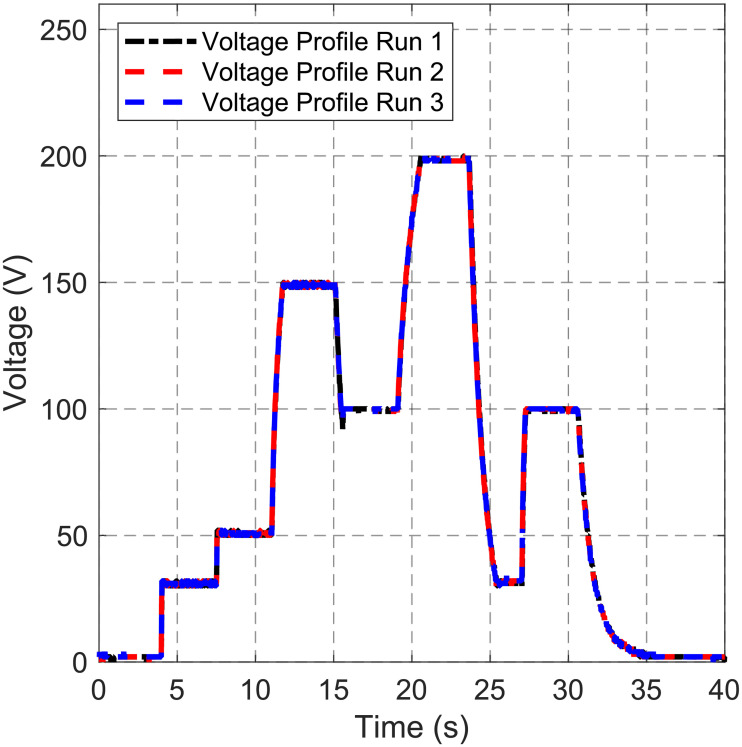
Boost converter output voltage. A predefined voltage profile is consequently transferred to the voltage regulator microcontroller. The output voltage of the boost converter is depicted for 3 runs.

The boost converter voltage profile is given by a recipe in the software of the PortaDrop system. The desired voltages are 30 V, 50 V, 150 V, 100 V, 200 V, 30 V, 100 V, respectively and a voltage turn-off at the end of the experiment. The boost converter runs at 9 kHz frequency of the PWM signal and the duty cycle is controlled by the voltage regulator microcontroller ATtiny45. Fig 7 shows overlaid transient voltage measurements of three runs recorded by a Tektronix TDS2024B oscilloscope (Tektronix, Beaverton, OR, USA). It is worth to denote that the input of the oscilloscope represents an additional load, which lowers the measured dynamic performance compared to the performance without monitoring the output.

The voltage set point is quickly reached and hold until it is assumed to be stable. No overshoot can be observed during charging processes, while a small spike occurs for discharging to the set point of 100 V. All three curves exhibit the identical time behavior.

Beside the use of the internal boost converter, an external high-voltage device can be connected to the PortaDrop system and is used to characterize the rectifier circuit. For this purpose, the input of the H-bridge is automatically connected via relay switching controlled by the software. The output of the H-bridge operating at 10 Hz and input voltages between 50 V and 200 V is presented in S25 Fig in S1 File. Measurements are taken by a Tektronix TDS2024B oscilloscope.

**Fig 8:**
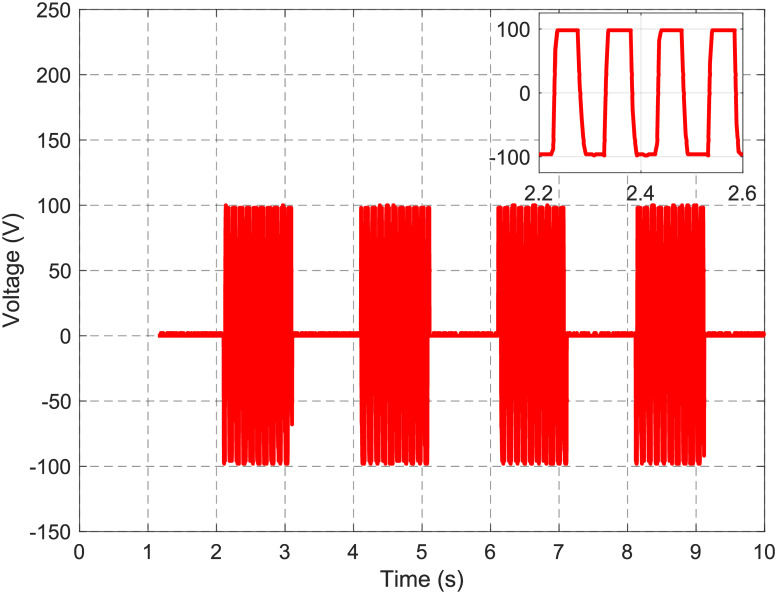
Voltage profile of a specific channel. The voltage amplitude is adjusted to 100 V with a frequency of 10 Hz and the channel is turned on and off with an interval of 1 s. The inset depicts a magnification of one period clearly showing the applied frequency and voltage amplitude.

In combination, the H-bridge, the internal boost converter and the semiconductor based pull-down and pull-up networks provide the ability to individually address path electrodes to enable droplet actuation by means of EWOD. The boost converter set point is set to 100 V, the frequency of the H-bridge to 10 Hz. The oscilloscope is directly connected to AC- and to one channel of the 60-lead ribbon cable, which is in the latter application directly connected to the path electrode. The channel is switched on and off for 1 s, respectively. Fig 8 depicts the switching pattern.

While the channel is deactivated, the pull-down network connects the channel with AC- and the voltage difference is 0 V. Upon activation of the pad, the output of the boost converter chopped into a bipolar rectangle signal by the H-bridge can be seen in the measurement. The inset in Fig 8 points out that the channel is powered with the desired amplitude and frequency. An additional voltage profile with different amplitudes delivered to the channel is shown in S26 Fig in S1 File.

For droplet actuation, the internally generated bipolar high-voltage signal is used. Therefore, a bottom chip with path electrodes made of gold, a dielectric layer of 4.95 μm thick Parylene-C and a cover layer of 50 nm thick Teflon were fabricated according the process as described by Pollack et al. [27]. The utilized top chip consists of an ITO ground plane, which is patterned by lift-off techniques. In addition, gold interdigitated electrodes (IDEs) are integrated in a round opening in the ITO ground electrode using standard UV-lithography and lift-off techniques adapting the idea of Eichler et al. [85]. IDEs with width and gap designed of 5 μm each were circularly arranged. A microscope photo of the electrodes is provided in S27 Fig in S1 File. After electrode deposition, the top chip is coated with a thin film of Teflon, masked with a photoresist and consequently patterned by means of reactive ion etching in oxygen plasma for opening the sensor spot [86]. This chip design allows not only droplet actuation but at the same time EIS measurements in the virtual well.

**Fig 9:**
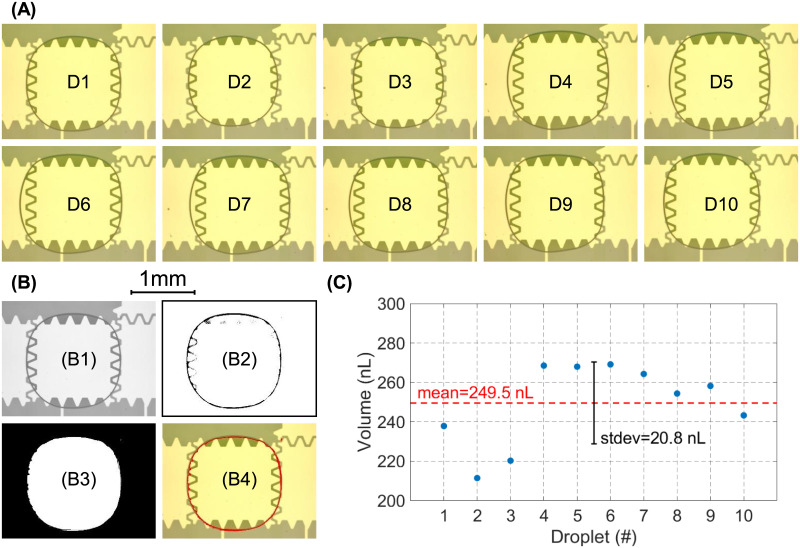
Droplet dispensing accuracy. (A) Microscope images of 10 individually dispensed droplets resting on pad 25 (B) Image processing procedure exemplary on droplet D1. The images are first converted to grayscale (B1), a threshold filtering is applied to amplify the edges (B2) and the area outside and inside the droplet is converted to black or white, respectively (B3). (B4) depicts the overlay of the droplet boundary (red) and the original image. (C) Derived droplet volume for droplet D1-D10 including mean value and standard deviation.

As a proof of the fluid handling capabilities of PortaDrop, droplets of deionized water were first actively dispensed from a reservoir and the volume was calculated by image processing. A 10 Hz signal with an amplitude of 200 V was generated using the boost converter and the H-bridge and consequently applied to the path electrodes by the semiconductor switches. Unit droplets, which cover one path electrode, have been dispensed from reservoir R2 (compare S16 Fig in S1 File) by first activating pad 25, 22 and 23, which pulls the liquid out of the reservoir. Subsequently, voltage was applied to pad 25, 23 and 20 to split the droplet (pad 22 remains in off-state). After one dispensing process, microscope pictures of the dispensed unit droplet have been taken. The procedure has been repeated in total 10 times. Fig 9 shows all individually dispensed droplets (D1-D10) as well as the image processing procedure and the derived droplet volumes.

Investigated with naked eye, droplet dispensing seems to be very reliable. As expected, due to the actuation pattern, the unit droplet tears off on pad 25. For detailed investigation a custom-made MATLAB image processing script was utilized (Fig 9(B)), where the image was first converted to grayscale, threshold filtered to amplify the droplet boundaries and converted to a logical array, where black (false) represents the area outside the droplet and white (true) the droplet. For verification, the obtained boundary was superimposed with the original image. The calculation of the area covered with fluid was carried out by counting the white pixels in the black and white image (B3). Finally, the number of pixels was scaled to a physical area by scaling with a known dimension in terms of the edge length of the underlying path electrode. For volume calculation, the obtained area was multiplied with the thickness of the spacer between bottom and top chip of 130 μm. Fig 9(C) depicts the volume of every single droplet, ranging from 211 nL to 269 nL. The arithmetic mean was determined to be 249.5 nL and the standard deviation to 20.8 nL, respectively.

**Fig 10:**
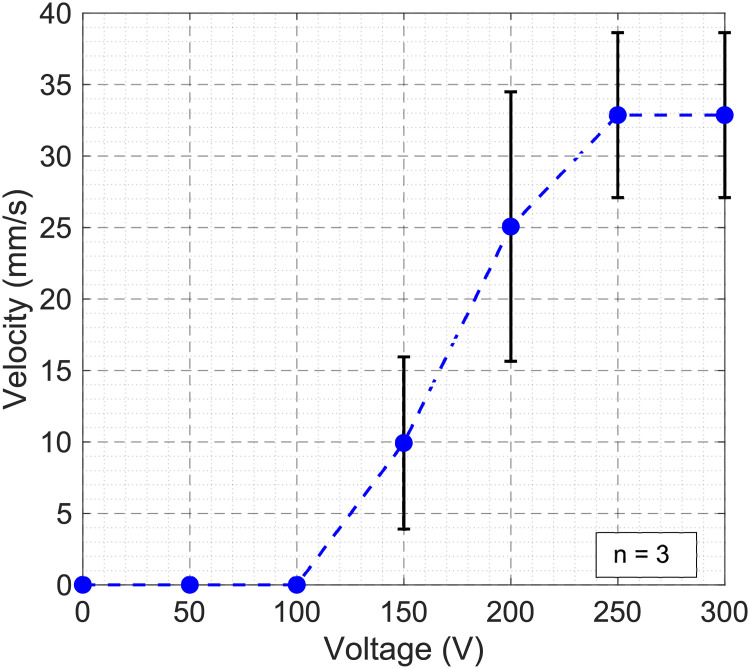
Droplet velocity. Maximum average droplet velocity with respect to the applied voltage.

Another important need for DMF systems is the ability to move droplets sufficiently fast across the chip. Therefore, PortaDrop was utilized to move droplets of deionized water across the device and the maximum average droplet velocity was derived. Therefore, a 10 Hz signal of different voltage amplitudes was generated. The average velocity was obtained by decreasing the switched-on time of each path electrode until the droplet is not able to follow anymore. Droplet actuation was counted as successful when the droplet passed through a figure eight track including 24 path electrodes (electrode length = 1300 μm, electrode spacing = 40 μm). The entire experiment was carried out three times. Between the experiments, the chips were unmounted from PortaDrop and dried to obtain new and random alignment of the chips for each repetition. Fig 10 depicts the mean droplet velocity with respect to the applied voltage and the standard deviation.

For low voltages, the velocity is zero and increases above a certain threshold value. At high voltages above 250 V, no more increase in droplet speed is observable. The experiment-to-experiment deviations are due to locally different surface conditions. For most applications, the droplet speed is not crucial as long it is sufficiently high and reliable droplet motion can be ensured. More important is the sample dispensing accuracy (Fig 9) and the latter discussed accuracy of EIS measurements. A video of an experiment is provided in S2 Video. Here, the droplet is first dispensed from a reservoir and then moved around the described figure eight track 15 times at an average speed of 26.8 mm/s.

**Fig 11:**
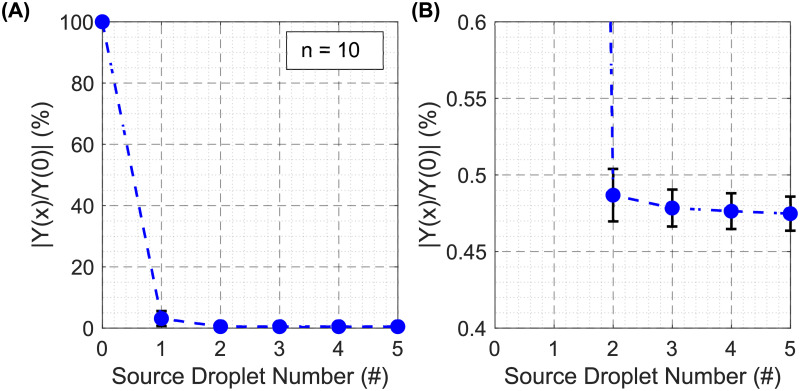
EIS measurements during passive dispensing. (A) Course of the electrical admittance at 205 kHz with respect to the number of the droplet moved across the EIS sensor normalized to droplet 0 (PBS). The droplet numbers 1 to 5 correspond to consequently applied DI water droplets. (B) Zoom plot to visualize the small deviations after the second exchange droplet.

To demonstrate the ability of PortaDrop to provide electrochemical measurements, the exchange of media by passive dispensing is monitored. A droplet of phosphate-buffered solution (PBS) with a pH value of 7.4 and a conductivity of 1.6 S/m is dispensed from one reservoir and moved across the sensor, where the droplet tears-off due to the difference in hydrophobicity by building a column underneath the sensor, called virtual microwell [71]. The electrical impedance is then measured by EIS. Consequently, five deionized (DI) water source droplets are dispensed from a second reservoir and moved across the sensor as well, diluting the content of the virtual microwell. After every droplet passage, a full spectrum of the electrical impedance is taken between 50 Hz and 1 MHz. The obtained spectra of the first experiment are shown exemplary in S28 Fig in S1 File. The normalized course of the electrical admittance Y at 205 kHz is depicted in Fig 11. 100% represents the conductivity of the initially delivered PBS droplet. Blue points represent the arithmetic mean values (n = 10) and the error bars the standard deviation.

Due to every DI water droplet moved across the sensor, the content of the virtual microwell is diluted, as can be observed by a decrease in the admittance. The decrease is most significant when the first droplet of DI water passes the sensor. After passing of droplet number 3, the course of the normalized admittance does not change significantly anymore. A video of the experiment including the droplet actuation and the impedance measurements is provided in S3 Video.

## Discussion

The portable all-in-one box DMF PortaDrop system is presented. For its operation, only one common 5 V USB power supply is necessary. The PortaDrop comes with internal functionalities for (i) droplet transport and (ii) an internal EmStat pico potentiostat for various electrochemical sensing application. In the first version, only EIS measurements are implemented. Additional electrochemical protocols provided by the EmStat pico can be easily implemented and make use of the infrastructure on the mainboard, e.g. routing capabilities to set up the electrode configuration (2-, 3- or 4-wire configuration). Compared to portable EWOD systems described before [34, 36, 37, 40, 43, 44, 46, 47] PortaDrop is unique by using a Raspberry Pi and thus a Linux-based operating systems. Beside others, this feature provides the advantage of an easy adaption to a LAN environment as well as to the external devices. The USB ports of the Raspberry Pi offer the beneficial possibility to connect different instruments by plug-n-play. For example, one GPIB-to-USB Adapter can control up to 15 devices at the same time. Of course, keyboard and mouse as well as external monitors can be connected to carry out experiments with PortaDrop more comfortable.

A simple mechanical interface for interconnection of DMF chip and electronic board is needed to guarantee easy to handle operation and management of the reservoirs. For alignment of bottom and top chip, a 3D-printed fixture is presented (see S24 Fig in S1 File). The adapter itself reduces the evaporation of droplets sandwiched between bottom and top chip. In general, the reduction of evaporation is beneficial for long-term experiments in DMF devices. For PortaDrop, 3D-printing can be used to implement different adapters and fixtures without the need of costly and time consuming micromachining processes, e.g. to implement capillary connections to the reservoirs for loading fluid from the outside as presented by Kim et al. [42]. The cable-connected adapter can be used in the all-in-one box but can also be adopted easily to a microscope or an external analytical device.

The implemented boost converter is controlled in a closed loop regulated by an ATtiny45 microcontroller (see Fig 3). The desired output voltage can be set in the GUI. Fig 7 depicts the output voltage of the boost converter for a pre-defined voltage profile. The set points are reached within a few seconds. It is obvious that charging to higher voltages takes more time since more charge needs to be delivered to the output capacitor of the boost converter. The time constant of the implemented proportional controller is significantly smaller compared to the RC charging time constant. This ensures that no voltage overshooting occurs, which might damage the EWOD chips due to dielectric breakdown of the insulating cover layers of the electrodes resulting in an irreversible device failure [24]. The boost converter is able to keep the voltage constant once the set point is reached without an observable ripple. Discharging of the boost converter is only due to the load of the path electrodes and the feedback resistors (30,3 MΩ). When a lower voltage is required during the recipe, the controller automatically discharges the output via a discharging network (see S3 Fig in S1 File). Here, an overshoot can be seen in Fig 7 around t = 15 s when discharging from 150 V to 100 V. This is due to the delay of the mechanical relay and is immediately balanced by the voltage regulator. It is worthwhile to mention that the oscilloscope, which was used for these measurements, adds an additional load to the system (input resistance 10 MΩ including divider). Without using the oscilloscope in normal operation, the set point will be reached faster. The profile is tested three time in a row, indicating very reproducible voltage regulation, which is beneficial for precise control of EWOD experiments (see Fig 7). The boost converter architecture is preferred due to the high efficiency and the low number of circuit elements.

For the generation of a bipolar high-voltage actuation signal, an H-bridge with optotriacs is utilized in PortaDrop (see Fig 4). The advantage of optotriacs is the galvanic decoupling of the high-voltage part from the TTL level on the microcontroller circuitry. The frequency is provided by an ATtiny45 microcontroller using a hardware timer with interrupt support maintaining the set frequency independent of the call stacks content. S25 Fig in S1 File shows the generated bipolar signals for different voltage amplitudes. All measurements concerning the H-bridge are carried out at only 10 Hz. This is because the oscilloscope and the voltage divider in series exhibit an input resistance of 10 MΩ and an input capacity of 18 pF leading to a time constant τ = RC = 0.18 ms. S29 Fig in S1 File depicts a voltage slope from 0 V to 100 V generated by the H-bridge revealing a measured time constant of around τ = 0.19 ms. Thus, the relatively high time constant is mainly defined by the load represented by the oscilloscope. Therefore, it can be assumed that much higher frequencies can be used during operation.

In combination with the pull-down and pull-up network (Fig 5), the developed circuitry is able to apply actuation signals to the path electrodes (see Fig 8) of the DMF bottom chip. Again, we use optoelectronics to realize fast switching between electrodes and ensuring high droplet velocity. It can be assumed that the droplet velocity is therefore only limited by inertia of the droplet and friction forces.

DMF platforms rely on the actuation of tiny droplets. Thus, the precision of dispensed droplets is from supreme importance. The average droplet volume was calculated to be 249.5 nL with a standard deviation of only 20.8 nL (n = 10). Since the utilized electrode geometry of the path electrodes is assigned to be a conventional geometry, certain variations in droplet volume occur. Nikapitiya et al. reported errors of up to 34% for these conventional designs while the volume of the dispensed droplets in our study is only 8.3% off the mean value [87]. They achieved higher accuracy by utilizing a TCC-shaped reservoir. It is worthy to denote that the optimization of the path electrodes was not the focus of the presented study.

The average droplet actuation speed was measured with respect to the applied voltage (see Fig 10). For droplet motion, a certain voltage is required to overcome friction forces [4]. Beyond the threshold, the velocity increases. The increase is ideally parabolic as described by the Young-Lippman equation [3]. At higher voltages, the effect of contact angle saturation arises [19]. A further increase of the voltage would lead to device failure by effects of air ionization or dielectric breakdown [32]. We were able to achieve a maximum average droplet velocity of 32.9 mm/s. The DropBot system presented by Fobel et al. has the advantage of the force and droplet position feedback [37]. It was shown that the peak velocity of a droplet can reach up to 80 mm/s while moving the droplet from one pad to the adjacent pad. It must be mentioned that in contrast to Fobel et al. the velocity was calculated as the average speed of a path across 24 pads with several 90° turns (l = 32.16 mm) in our experiment. Higher speeds were obtained using PortaDrop only locally where a droplet was able to partly follow the path of 24 pads at a speed of 53.6 mm/s (250 V, 100 Hz). This fact might be due to small variations in dielectric layer thickness or different conditions of the hydrophobic surface. In general, the surface condition of the Teflon layers is crucial. Small defect in terms of locally hydrophilic spots hamper the device performance. The standard deviation of 5.8 mm/s for the maximum average velocity might be due to different alignment of top and bottom chip in every experiment and thus different defects interfering with the droplets path.

As a first application, where the combination of EIS measurements with the outstanding fluid handling capabilities of PortaDrop show their ability of being a powerful micro total analysis system (μTAS) in future applications, the monitoring of the passive dispensing mechanism by EIS is presented. To our knowledge, this is the first time EIS was used to monitor the media exchange during passive dispensing in a virtual microwell. Passive dispensing is a well-known mechanism in DMF to allow precise fluid incubation underneath a hydrophilic spot in the top chip [14, 53, 55, 57, 71, 72]. Barbulovic-Nad et al. showed by fluorescence measurements that three source droplets are necessary to fully exchange the volume of the virtual microwell completely [14]. In our approach, EIS spectra have been recorded after every source droplet passing the sensor spot (see S28 Fig in S1 File and Fig 11). Since EIS is a label free non-invasive technique, the exchange from a PBS buffer to DI water can be monitored without any additives because of the invers proportional relation between electrical impedance and salt concentration in the media [88]. Our results obtained by EIS indicate that PortaDrop efficiently exchanges the content of the virtual microwell. After three exchange droplets, the spectra do not differ significantly from each other for further exchange droplets revealing that the media exchange is completed. The standard deviation of the admittance is below 0.02% after exchange droplet 2 to 5 showing a very good reproducibility. In the study of Barbulovic-Nad et al., at least three source droplets were necessary to replace the complete content of the virtual microwell [14]. Summarizing, our experiments demonstrate the powerful combination of EIS and DMF.

Compared to the DropBot approach in combination with the DStat, our presented system has the ability to carry out electrochemical measurements with on-board devices as well as external devices connected by coaxial cables when a higher frequency range is needed [37, 38]. In addition, PortaDrop provides one main software running on the Raspberry Pi, which is able to control all parts of the experiment.

The chip assembly and the cable-connected adapter allow to meet the requirements of individual applications of PortaDrop. The chip adapter on the top of the box can be redesigned. This allows for example an upright orientation of the chips. With path electrodes made from ITO instead of opaque chromium for example, experiments using a transmission approach can be carried out where light passes through the chip vertically. For optical spectra recordings, the Ocean Optics HR2000+ USB spectrometer is already implemented. In general, for the use of the fluid handling capabilities of PortaDrop by means of EWOD, only a connection to a 60-lead ribbon cable is necessary. This allows the use in e.g. incubators or climate chambers or the use underneath an upright light microscope. Electrochemical measurements can be easily performed. Therefore, a fixture concept was presented in the first version of PortaDrop. External devices can be used as well as the internal EmStat pico potentiostat.

## Conclusion

We presented PortaDrop, a portable all-in-one box DMF platform. The hard- and software as well as the assembly were introduced and the performance of the implemented components for bipolar high-voltage generation and path electrode switching were tested. The dispensing accuracy was investigated revealing highly reproducible droplet generation by means of EWOD within the PortaDrop system. Droplet transport using the EWOD effect has been demonstrated with a maximum droplet velocity of 32.9 mm/s for a track of 24 path electrodes. Locally, even higher velocities (53.6 mm/s) were obtained. The velocity can be further enhanced, e.g. by using a more uniform and smoother dielectric material. Switching times within the soft- and hardware implementation do not limit faster droplet actuation. To show the powerful combination of electrochemical measurements and EWOD, we monitored the media exchange in a virtual microwell due to passive dispensing by EIS for the first time showing that the complete content of the microwell is replaced after three exchange droplets. To summarize, we propose that PortaDrop will tackle the needs in different fields, where small volumes of analyte need to be handled and analyzed.

## Supporting information

S1 File (PDF)

S1 Experiment data (ZIP)

S1 Source code Source code of PortaDrop including a detailed readme for straight forward installation. Source code and readme are also available on GitHub: https://github.com/ewodac/PortaDrop.
(ZIP)

S1 Video Reservoir loading. (MP4)

S2 Video Droplet velocity test at v = 26.8 mm/s. (MP4)

S3 Video Passive dispensing and EIS. (MP4)
