# Supplementary material for: PortaDrop: A portable digital microfluidic platform providing versatile opportunities for Lab-On-A-Chip applications
Source: PLoS One. 2020 Sep 3;15(9):e0238581. doi: 10.1371/journal.pone.0238581 (PMC7470335; doi:10.1371/journal.pone.0238581)
Supplement: S1 File — (PDF) [file pone.0238581.s001.pdf]

Supporting information

PortaDrop: a portable digital microfluidic platform providing versatile opportunities for Lab-On-A-Chip applications

Tom Kremers[1], Sarah Thelen[1], Nils Bosbach[1], Uwe Schnakenberg[1]*

[1] Chair of Micro- and Nanosystems and Institute of Materials in Electrical Engineering 1, RWTH Aachen University, Aachen, Germany.

*Corresponding author

E-Mail: schnakenberg@iwe1.rwth-aachen.de

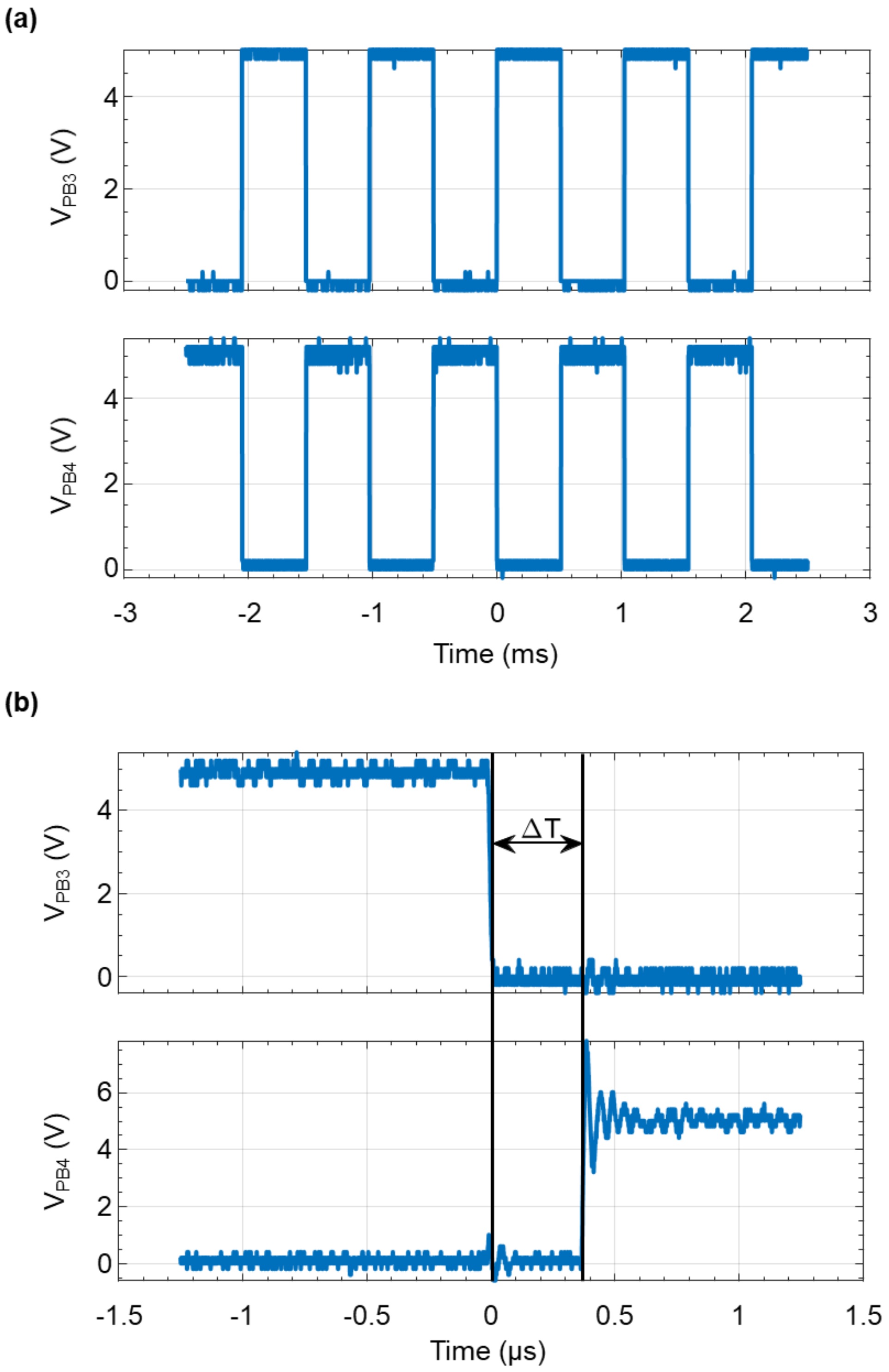

S1 Fig. Switching delay H-bridge. (a) 5 V rectangle signal at a frequency of 1 kHz and the inverted signal for the control of the H-bridge (b) Magnification of the change between HIGH and LOW and vice versa. The implemented delay corresponds to one processor cycle. For this time period, both signals are LOW and thus the connected optotriacs are turned off. Hence, a short between VOut and GND is avoided.

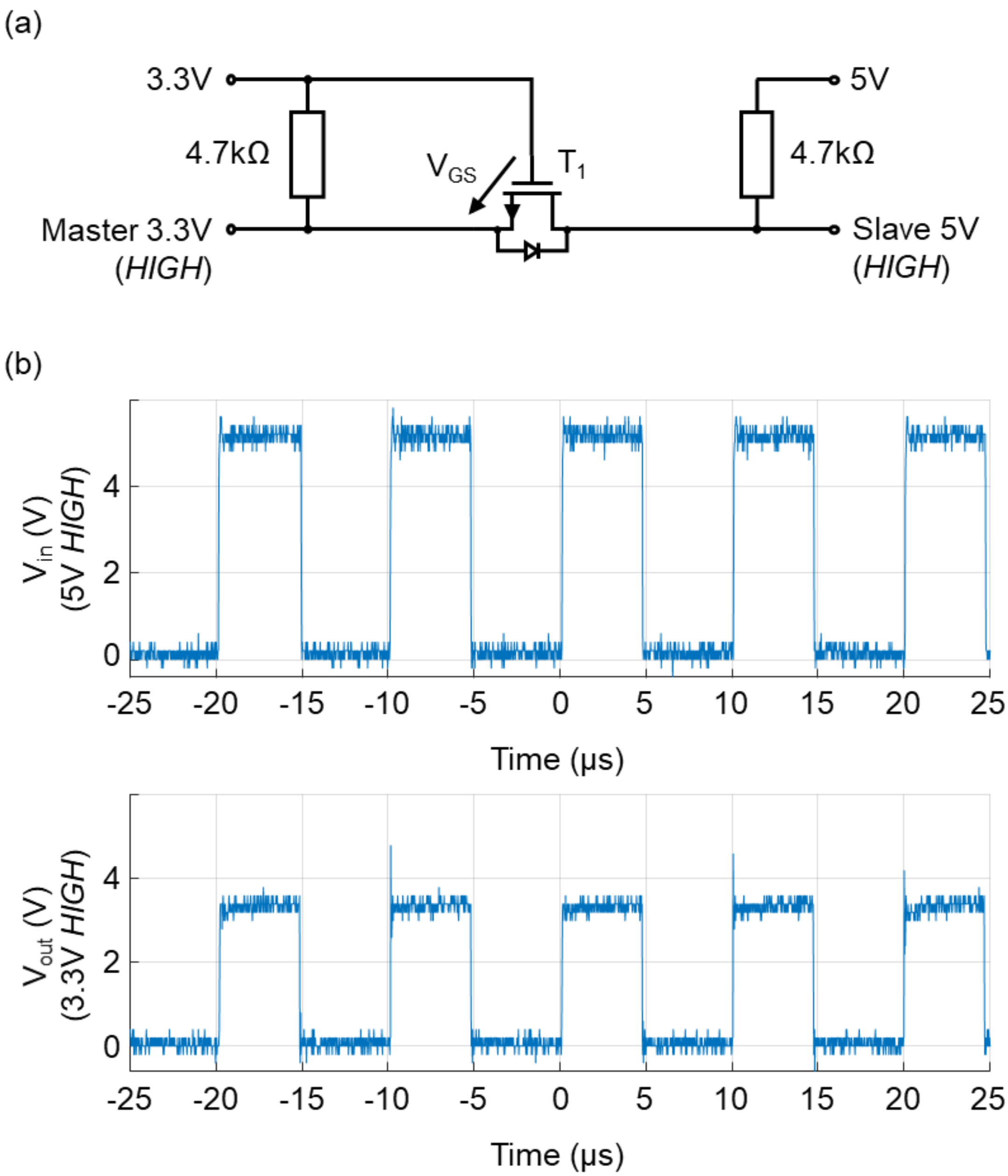

S2 Fig. I2C level shifter. a) Schematic of the I^2C level shifter. The Raspberry Pi operates with 3.3 V representing a logical *HIGH* and is the master of the I^2C bus while the AVR microcontrollers serving as the slaves detect 5 V as *HIGH* level. The level shifter is working bi-directional on the two wires of the I^2C bus SDA and SCL. b) Level conversion from 5 V to 3.3 V running at the standard I^2C frequency of 100 kHz.

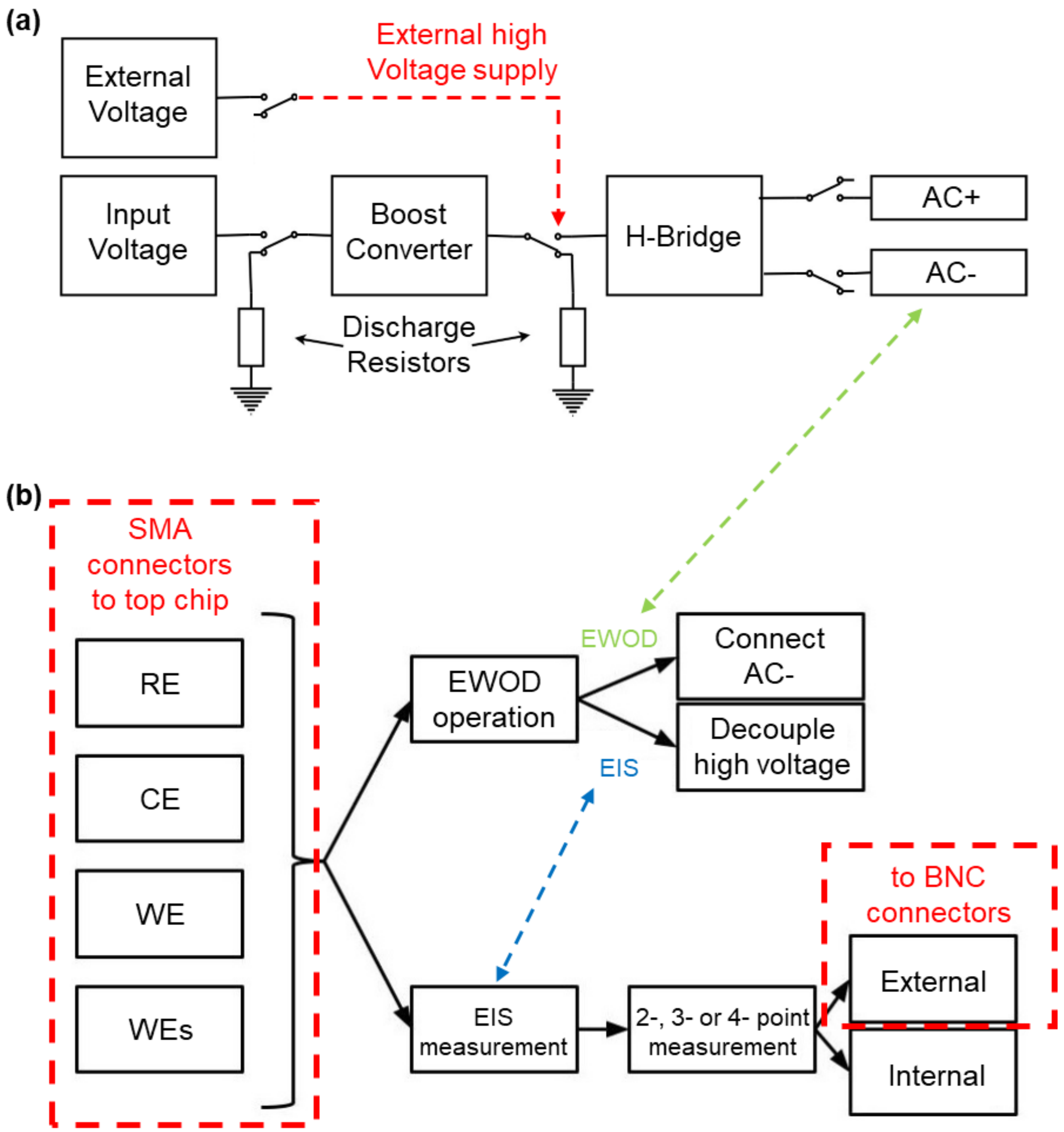

S3 Fig. Overview of the relays controlling the proper routing of the signals for EWOD and EIS operation. (a) The relays connected to the input and output of the boost converter disable or enable the boost converter, respectively. When the boost converter is enabled, the input terminal of the H-bridge is connected. In addition, an external voltage supply can be connected to the H-bridge. Two relays between the output of the H-bridge and the AC+ and AC- connectors allow to disconnect the voltage, thus the semiconductor switches boards are galvanically isolated. For EWOD operation both need to be connected to the blocks in (b), where the mode of operation can be switched from EWOD to EIS. Relays can therefore connect the electrodes on the top chip attached to the SMA connectors to the internal and external measurement devices and change the wire mode to 2-, 3- or 4-point measurement. At the same time the high-voltage is decoupled from the system to avoid damage of the devices as well as hydrolysis in the droplet.

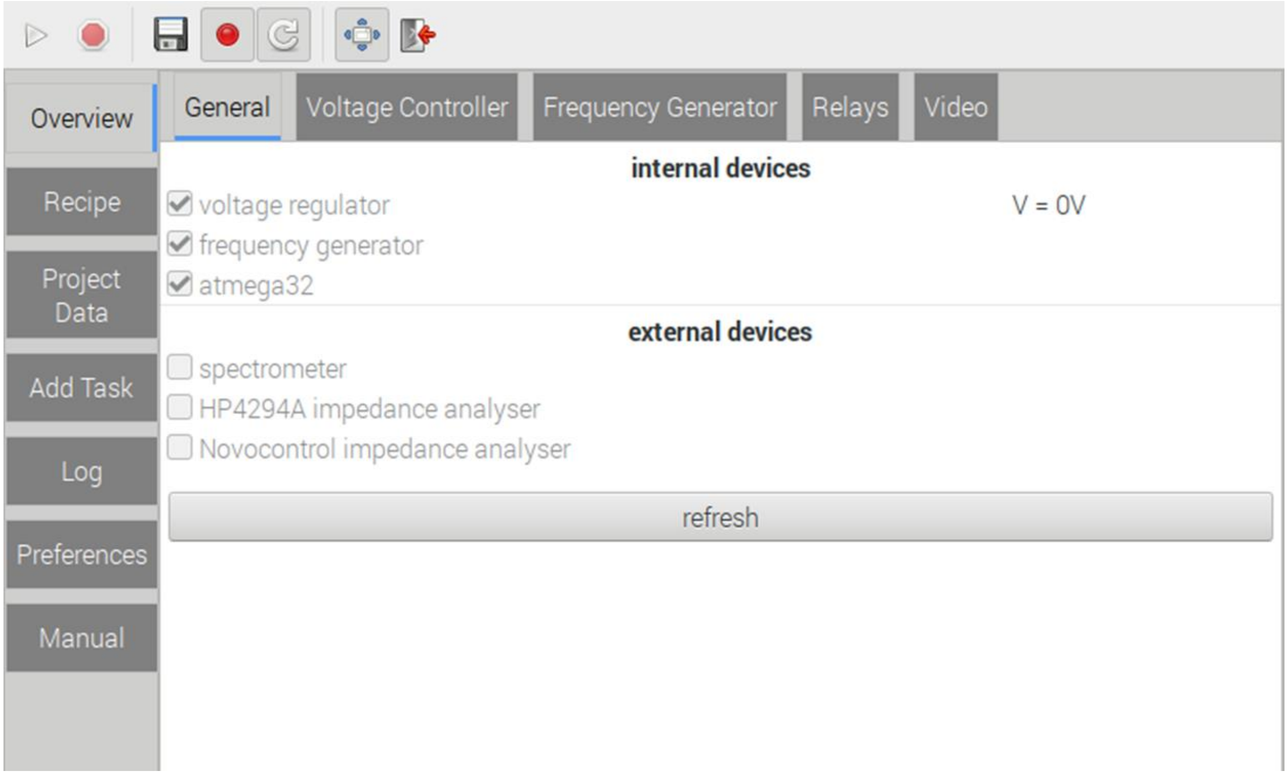

S4 Fig. GUI tab "Overview/General". The screen is providing information about connected external devices as well as the status of the communication via I^2C with the internal microcontrollers.

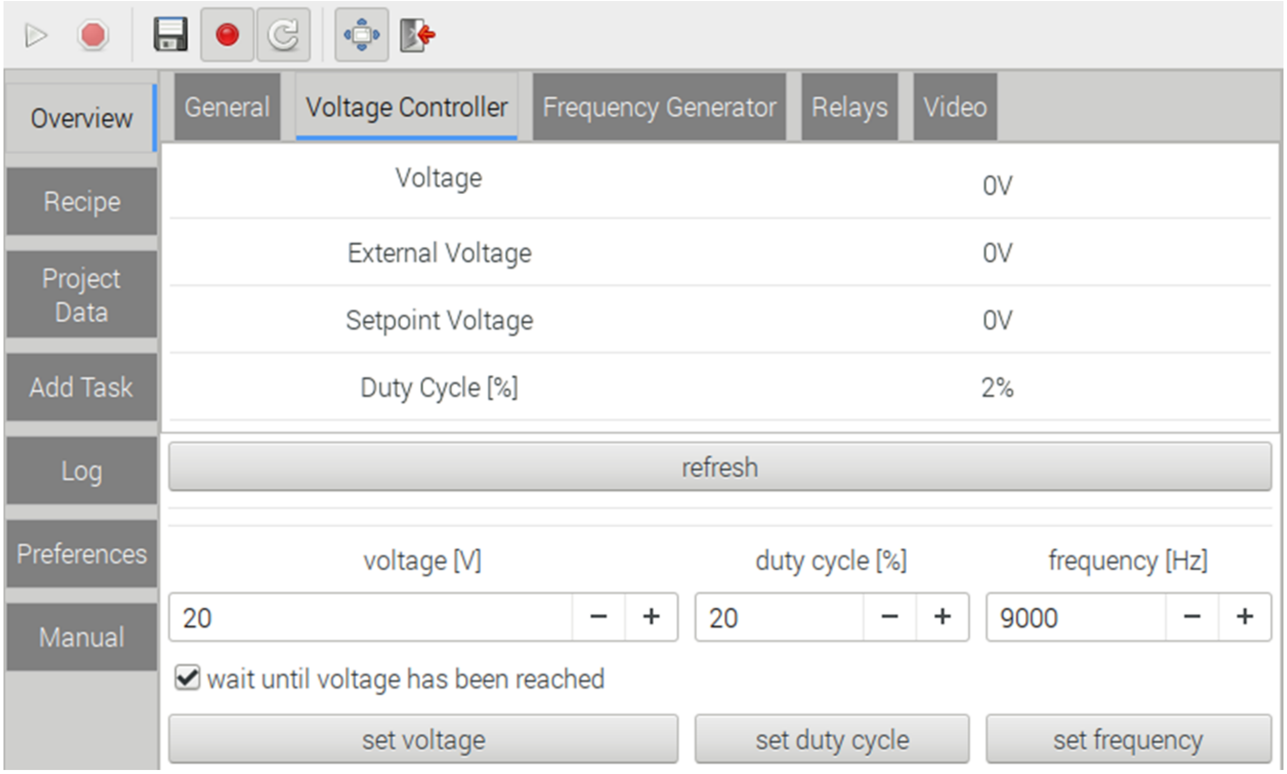

S5 Fig. GUI tab "Overview/Voltage Controller". The user is able to read out actual voltages and to send set points to the microcontroller. In addition, the frequency of the PWM signal can be set.

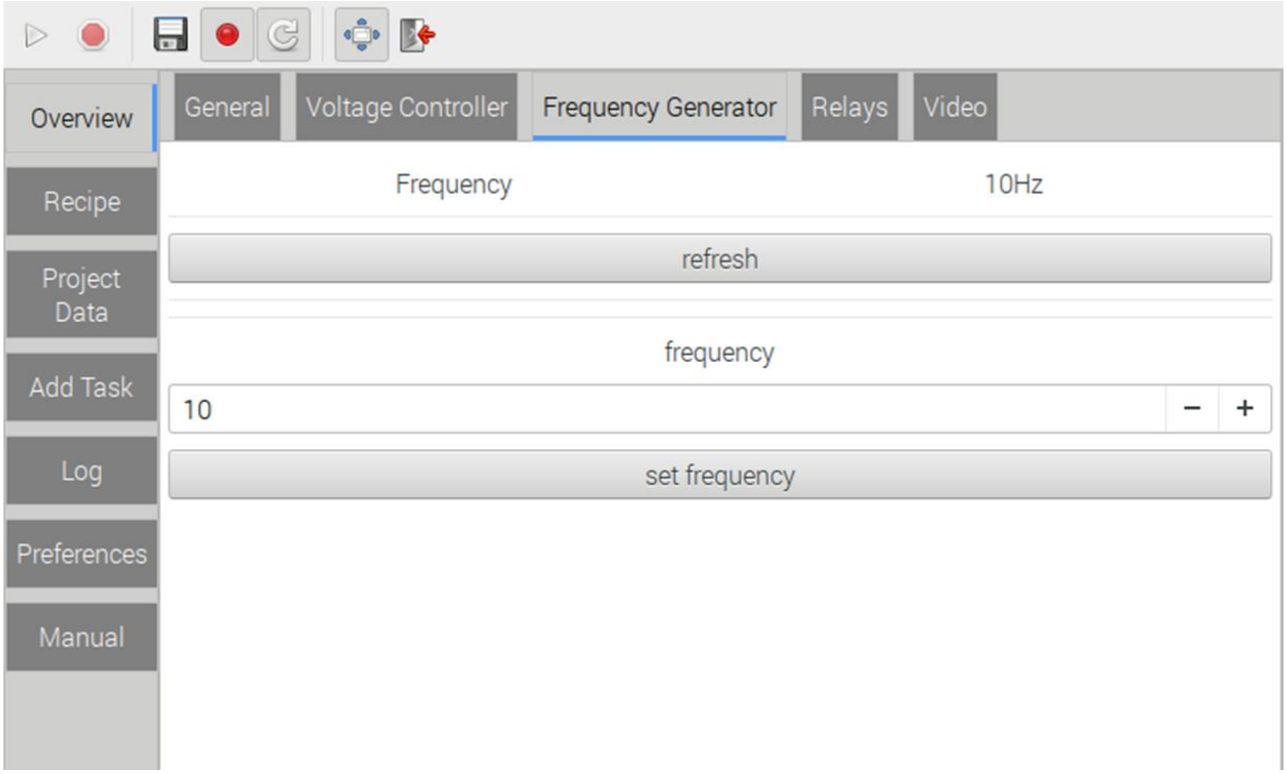

S6 Fig. GUI tab “Overview/Frequency Generator”. This page provides information about the current frequency of the rectifier circuit and allows to set a frequency manually.

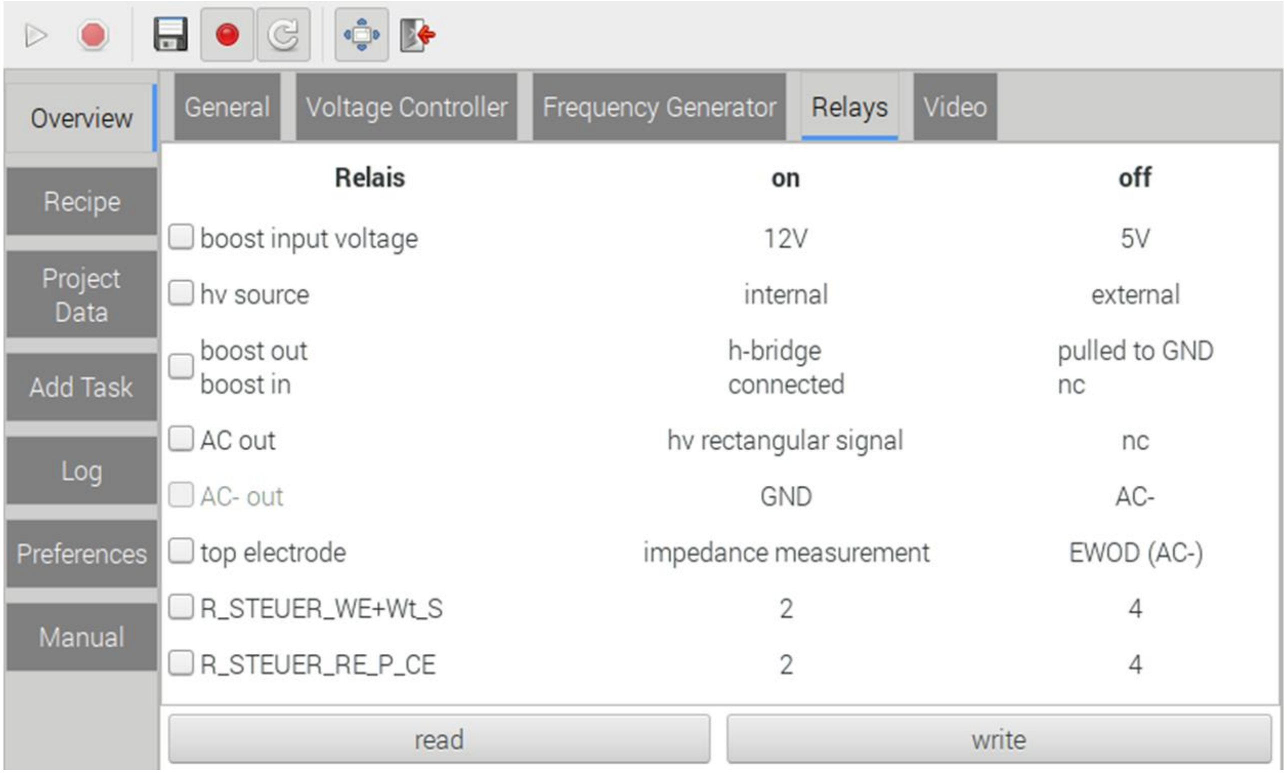

S7 Fig. GUI tab "Overview/Relays". The status of all relays on the mainboard can be seen. In addition, it is possible to un-/set the relays.

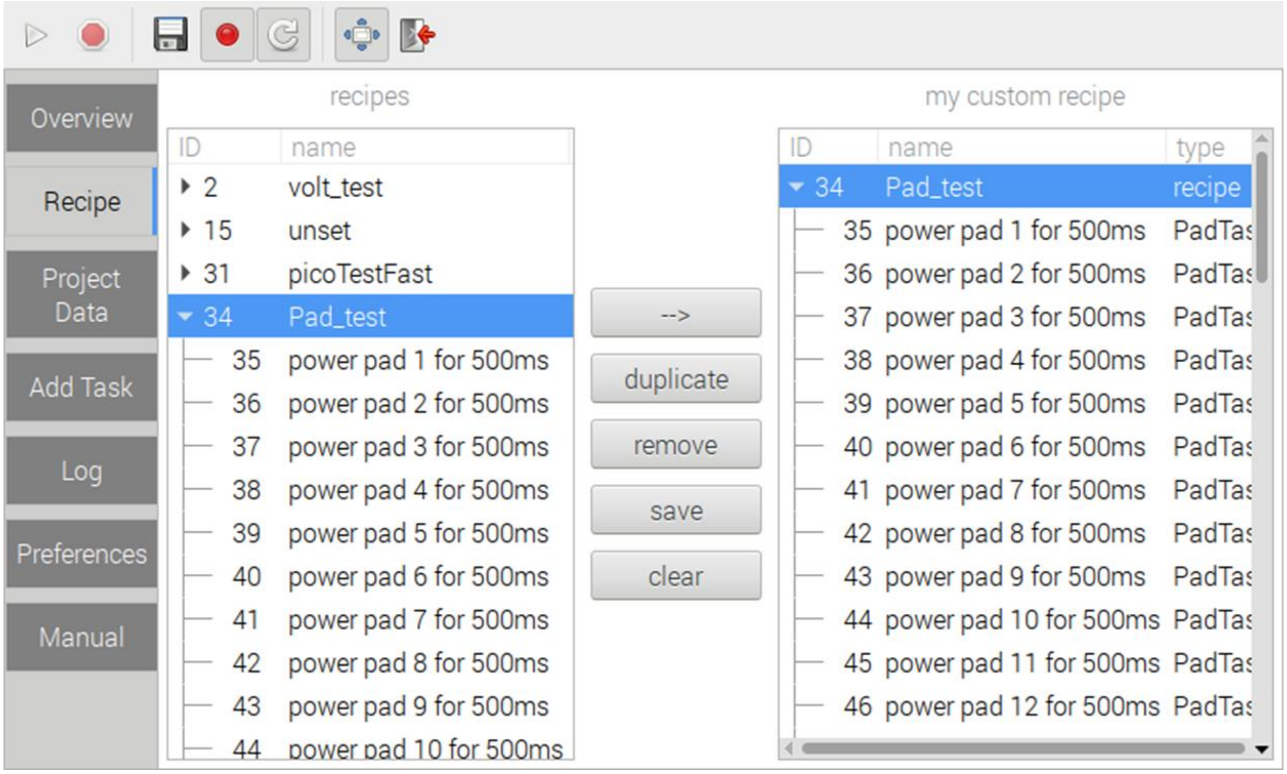

S8 Fig. GUI tab “Recipe”. The left column provides predefined recipes made from several steps. These recipes can be arranged to a custom recipe and saved. These custom recipes can serve as parts of future recipes as well.

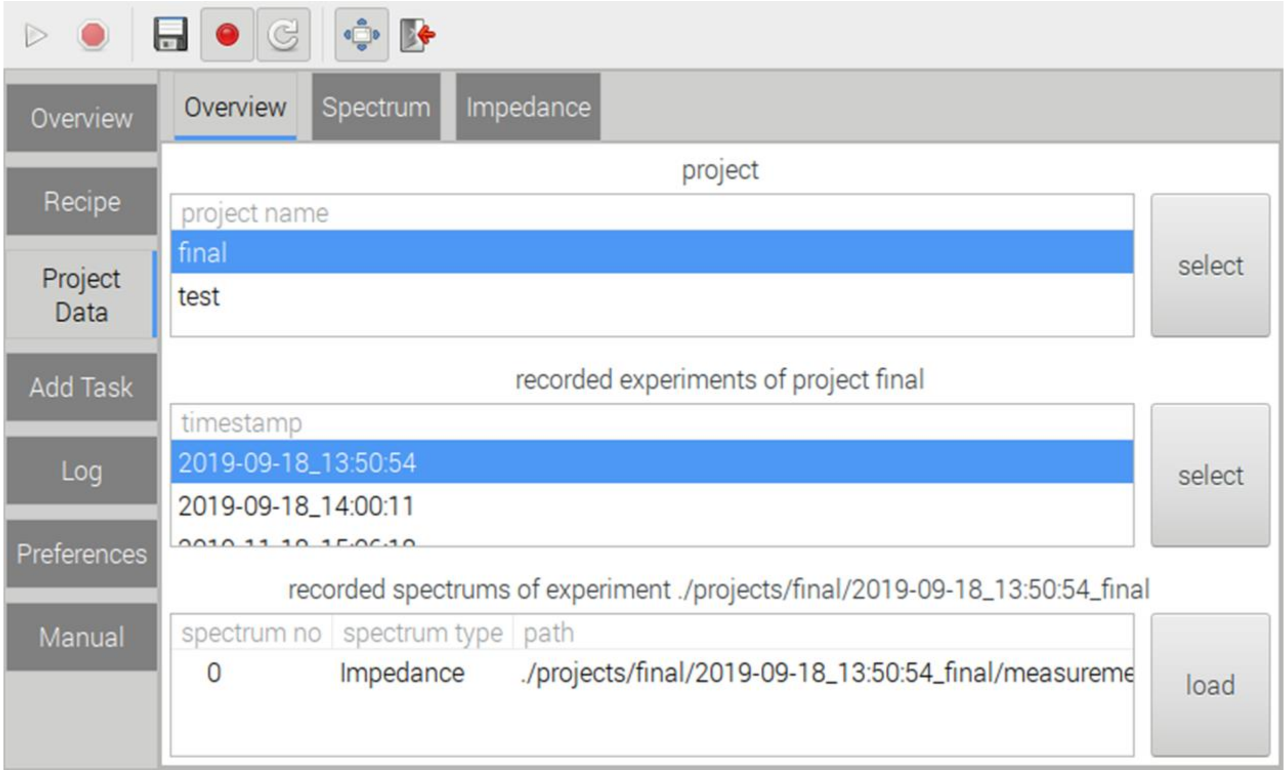

S9 Fig. GUI tab "Project Data/Overview". All experiments and measurements are saved in *.csv format. The GUI provides a section offering an overview about all recorded data.

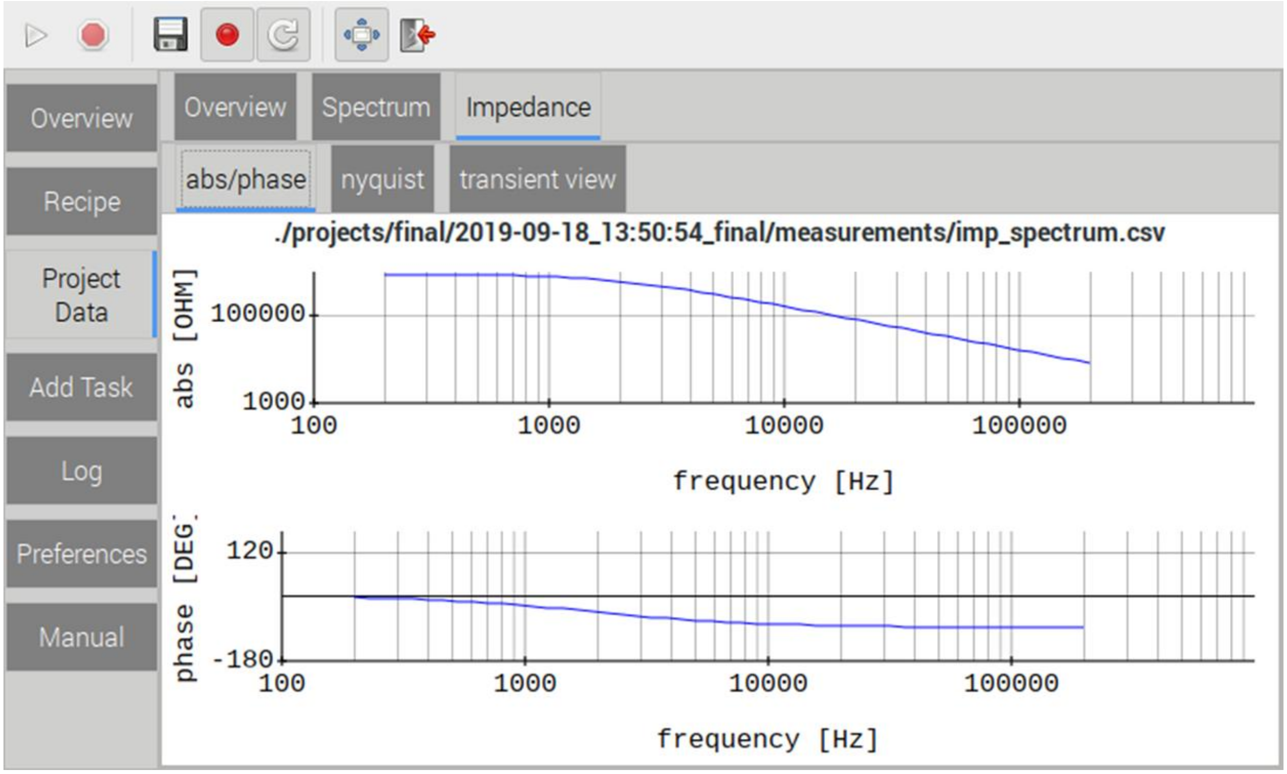

S10 Fig. GUI tab "Project Data/Impedance". During an experiment, EIS spectra can be depicted as a Bode plot, where magnitude and phase are plotted with respect to frequency.

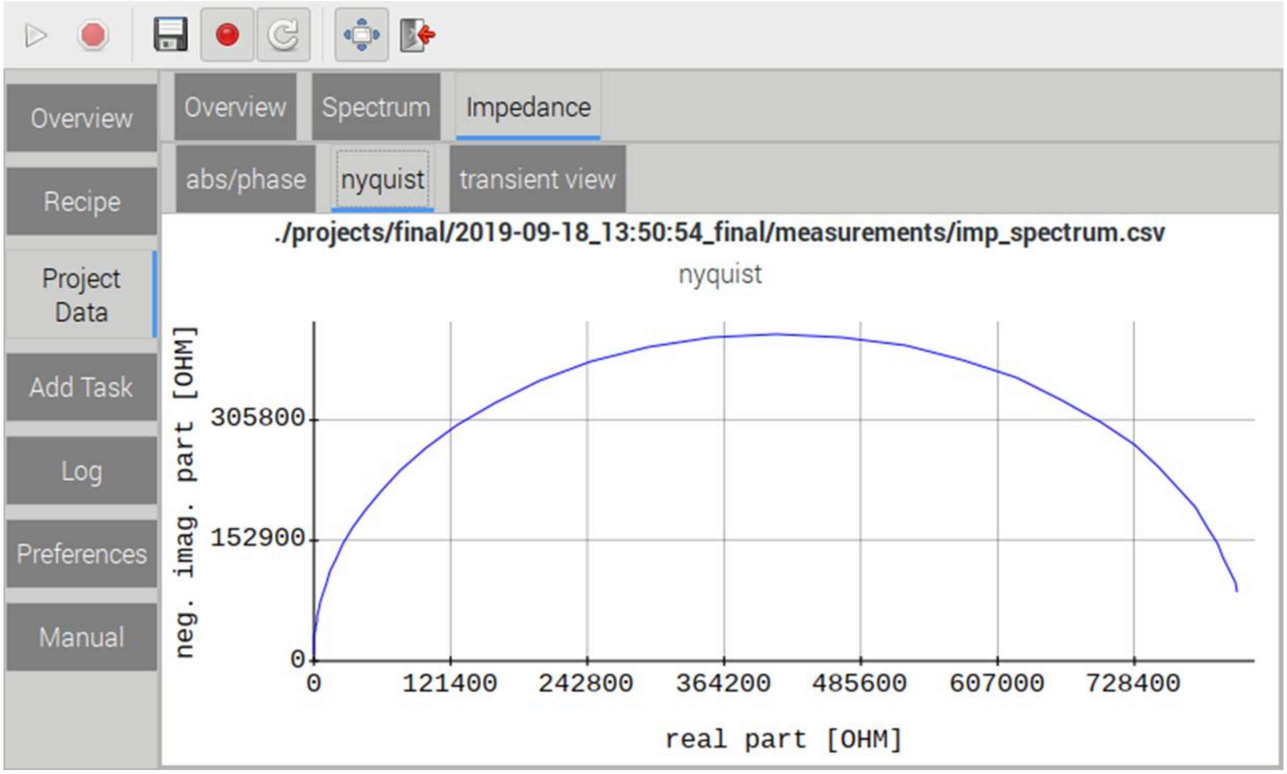

S11 Fig. GUI tab "Project Data/Impedance". In addition to Bode plots, the EIS data is shown in a Nyquist diagram.

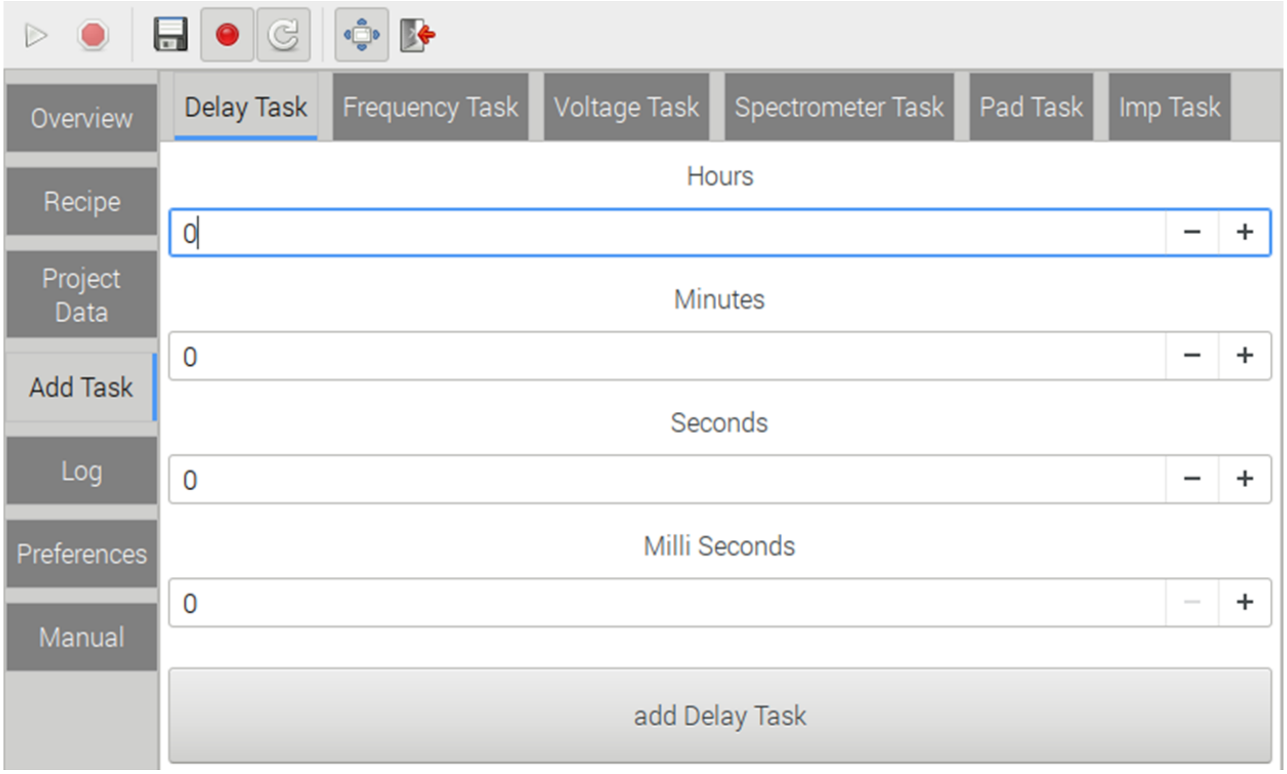

S12 Fig. GUI tab “Add Task/Delay Task”. Adds a delay to the recipe. During execution, no action occurs during that time period.

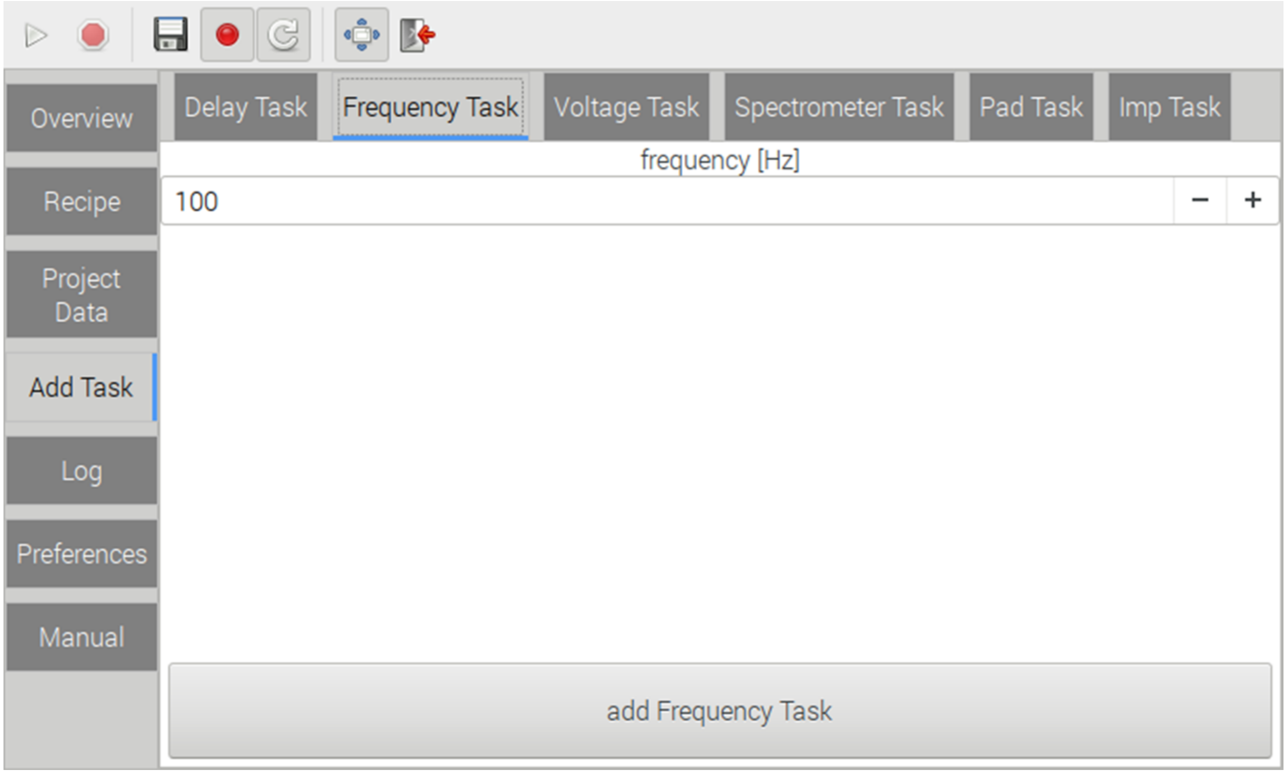

S13 Fig. GUI tab "Add Task/Frequency Task". A task activating the frequency generator (AC- and AC+ is connected) and setting the frequency to the desired value.

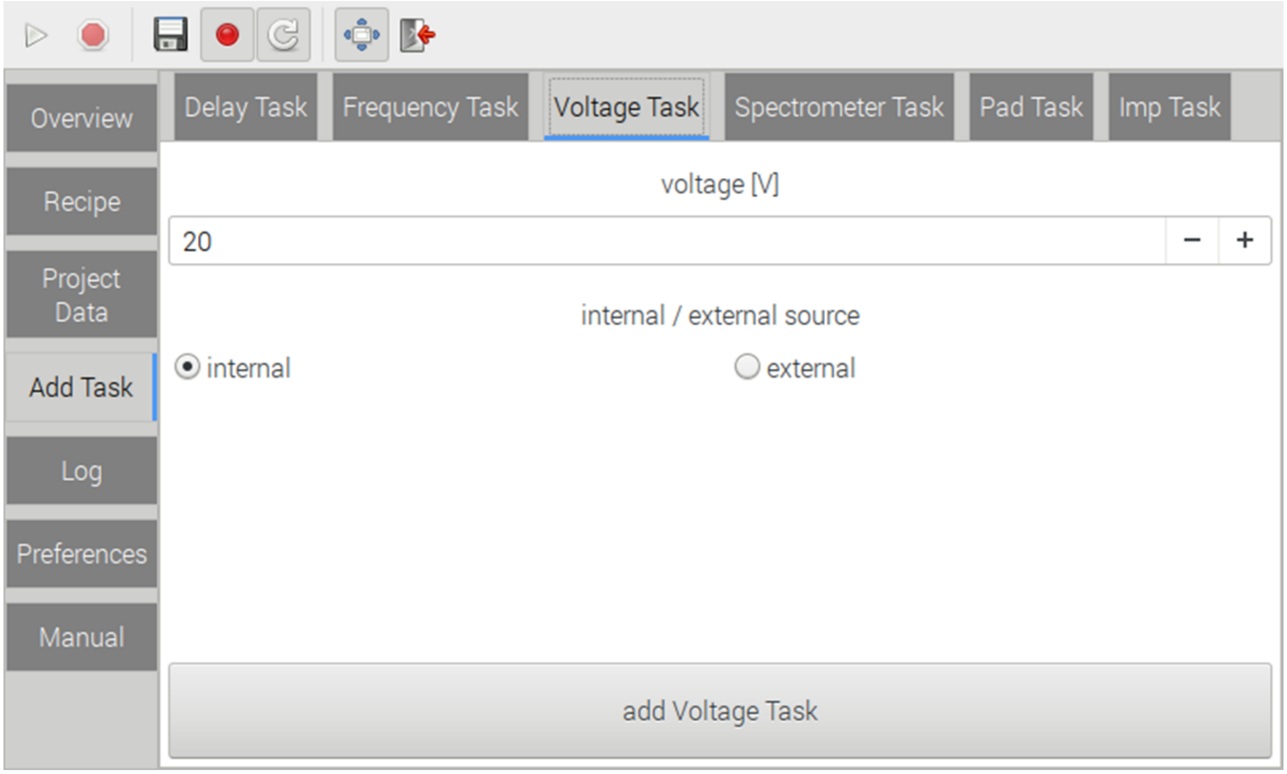

S14 Fig. GUI tab "Add Task/Voltage Task". Adds a voltage task to the current recipe. The voltage can be generated automatically using the boost converter (internal) or an external source can be used. During the execution, the user is asked to change the voltage to the defined set point.

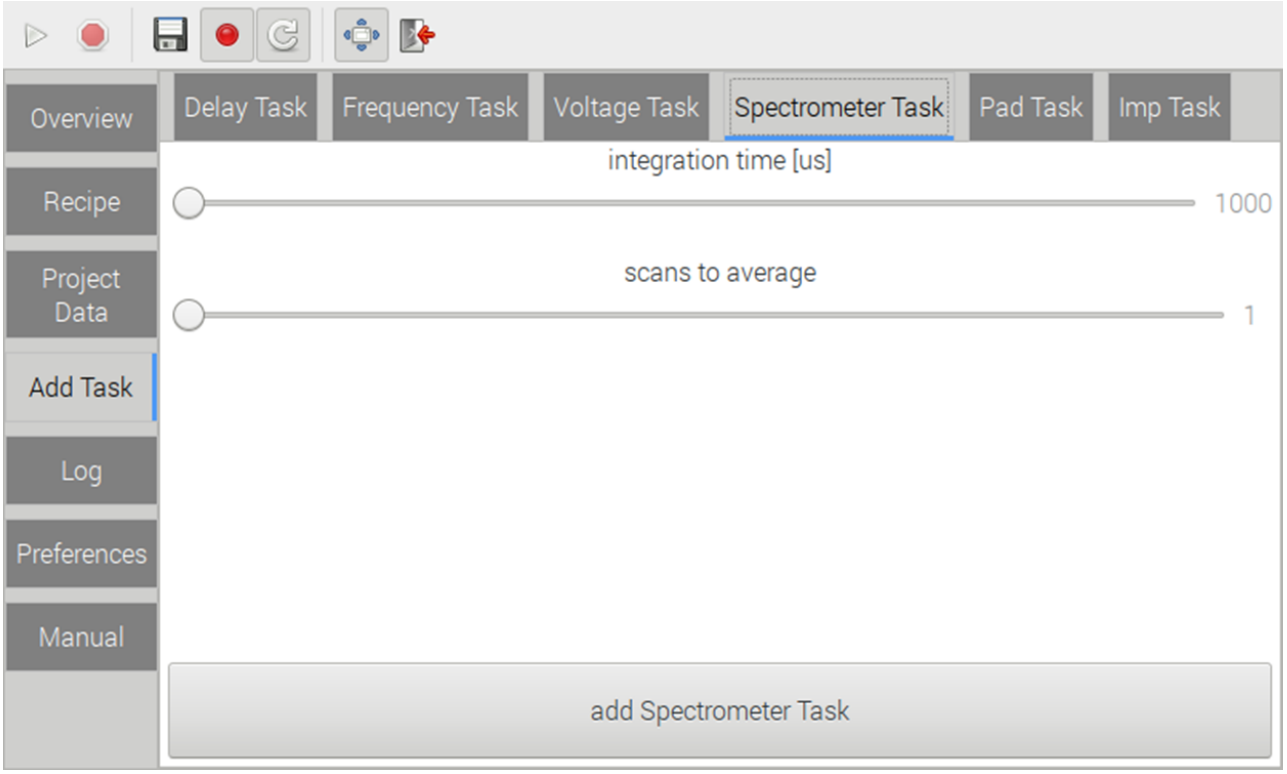

S15 Fig. GUI tab "Add Task/Spectrometer Task". Allows the use of the optical spectrometer (Ocean Optics HR2000+). The integration time per taken spectrum and the number of spectra for computing an average spectrum can be defined.

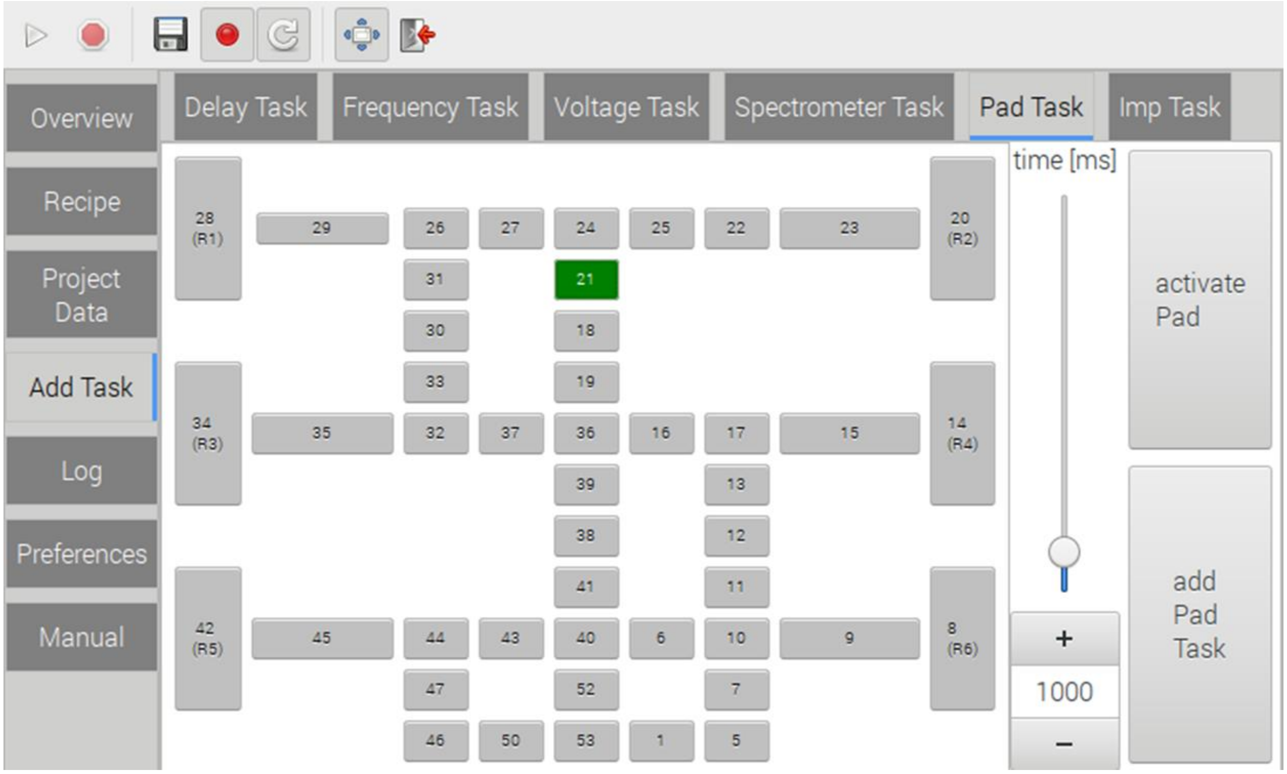

S16 Fig. GUI tab "Add Task/Pad Task". Overview of the used EWOD chip allowing to (1) immediately activate pads for the set time and (2) to add the task to the current recipe. The latter allows to specify sequences of activated path electrodes in a certain order.

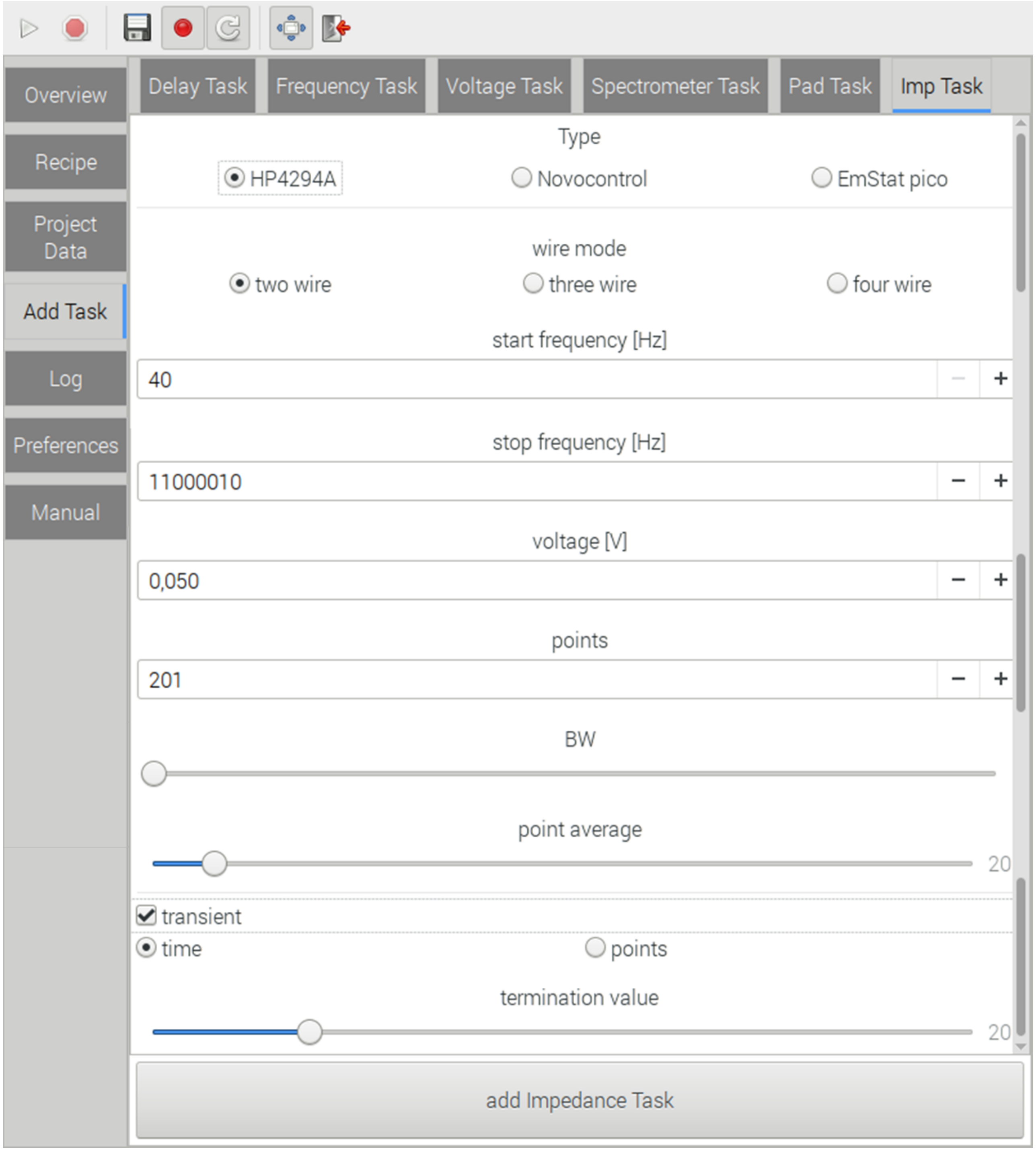

S17 Fig. GUI tab "Add Task/Imp Task". Settings of all EIS analyzers (extern via GPIB and intern) implemented can be set. During execution of a recipe, the software automatically takes care of the correct wiring and carries out the measurement specified here. Measurements are shown during execution of the recipe in the Project Data tab. The figure has been assembled to one large figure because the content exceeds the screen.

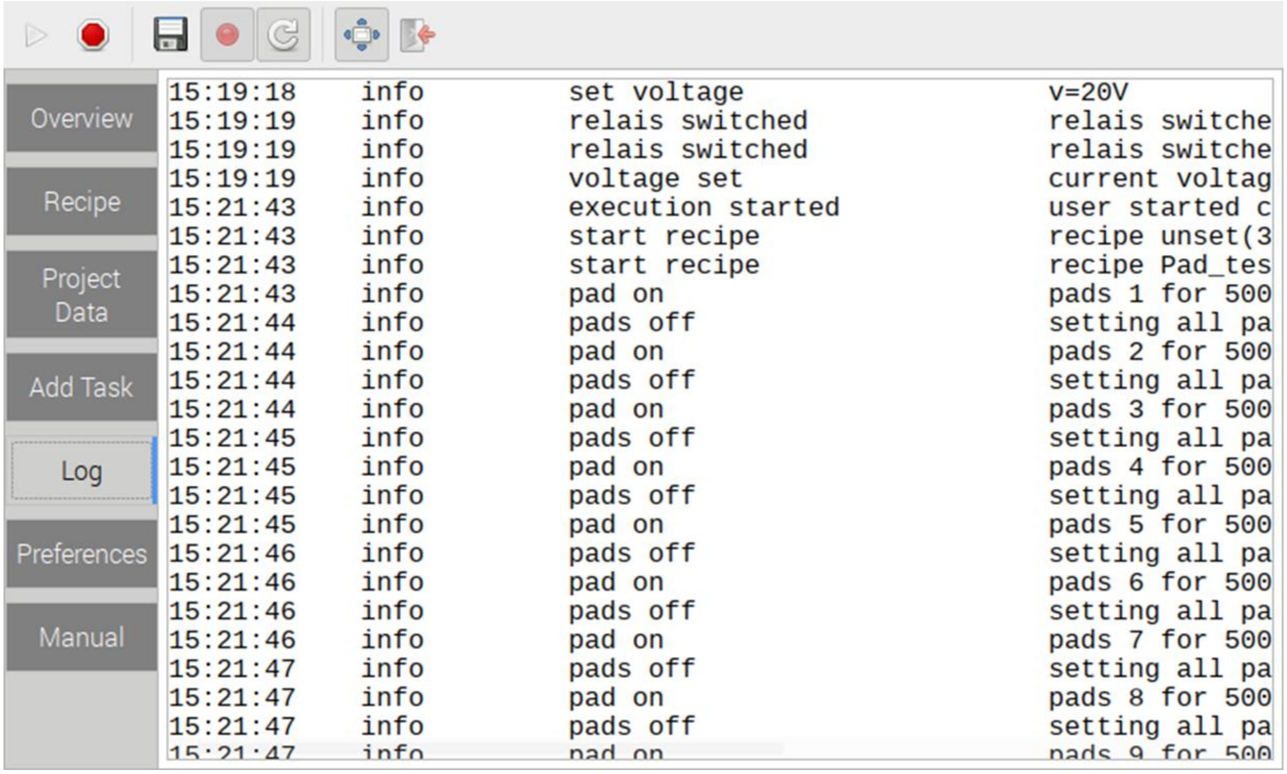

S18 Fig. GUI tab “Log”. Important events are stored during the entire execution of the software.

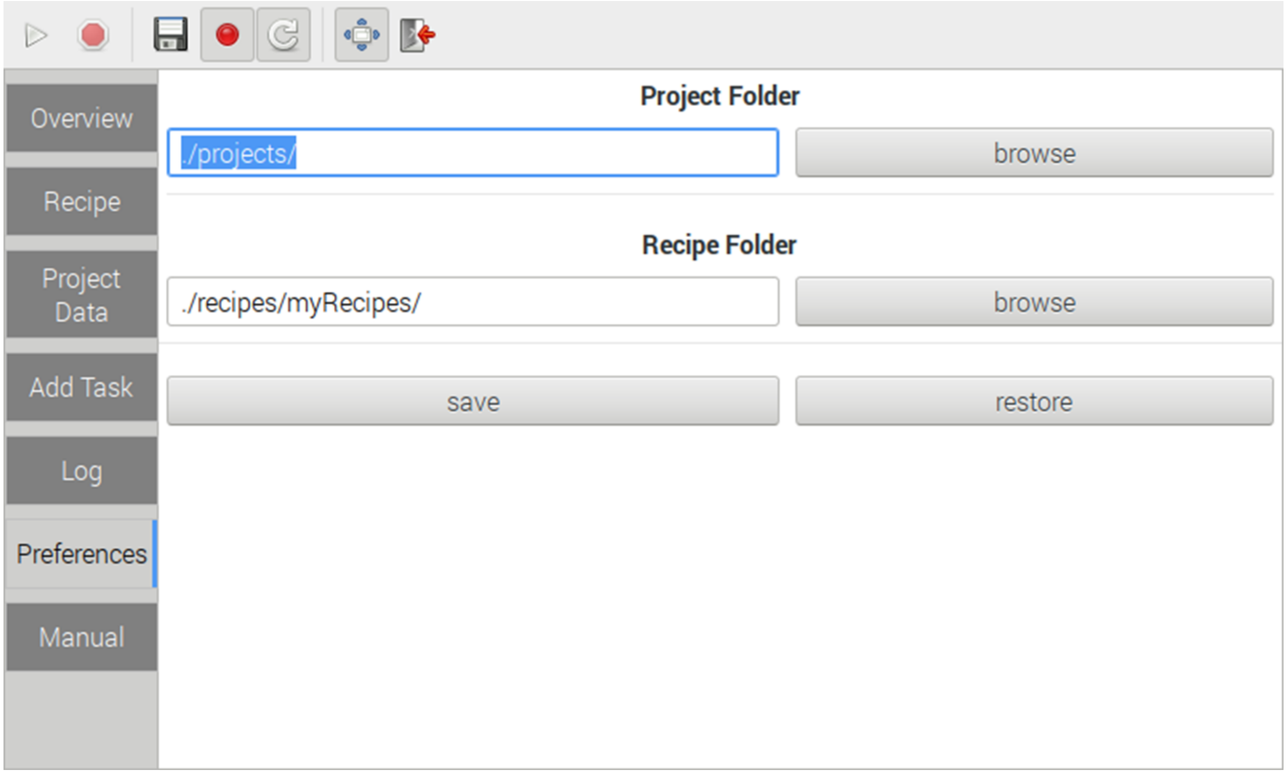

S19 Fig. GUI tab "Preferences". Path to the project and recipe folder.

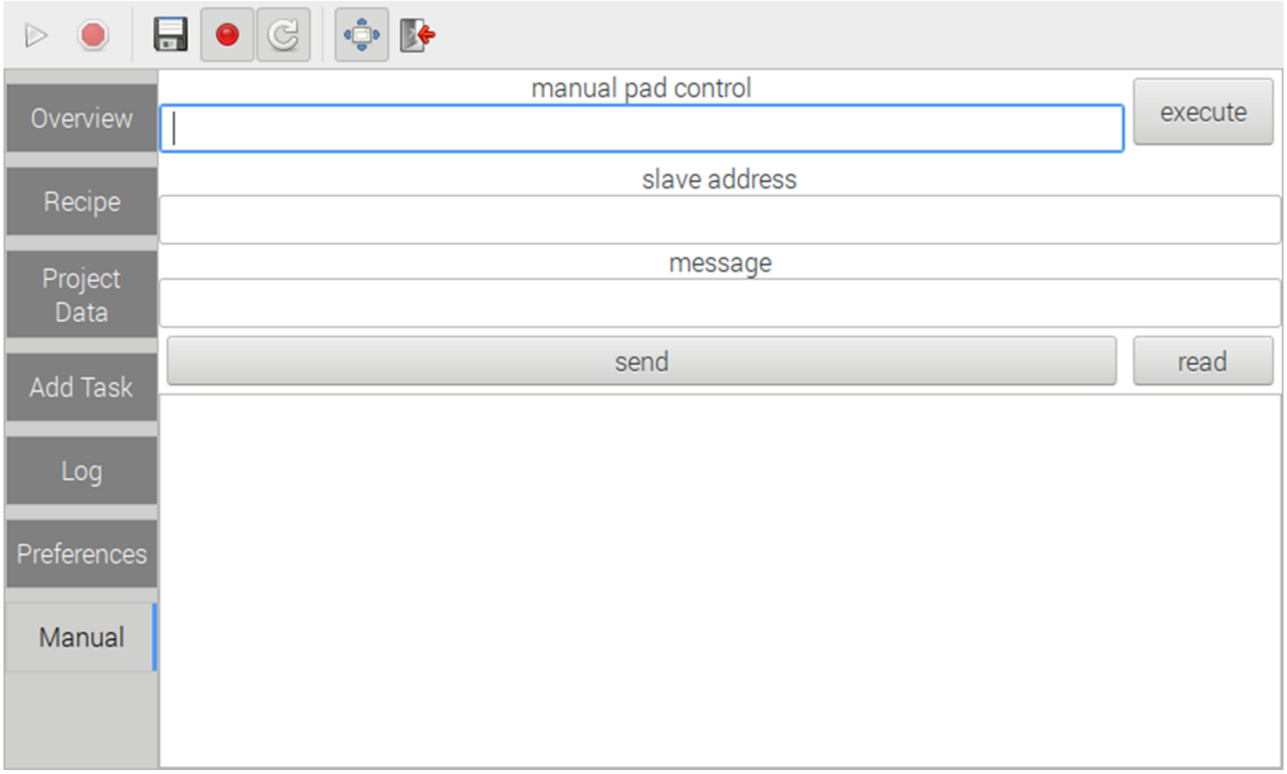

S20 Fig. GUI tab “Manual”. Comments for controlling the path electrodes can be send manually to the microcontrollers on the semiconductor switches board. In addition, GPIB addresses can be entered and GPIB comments can be send to the device and the reply is received. This tab serves for further addition of new GPIB devices.

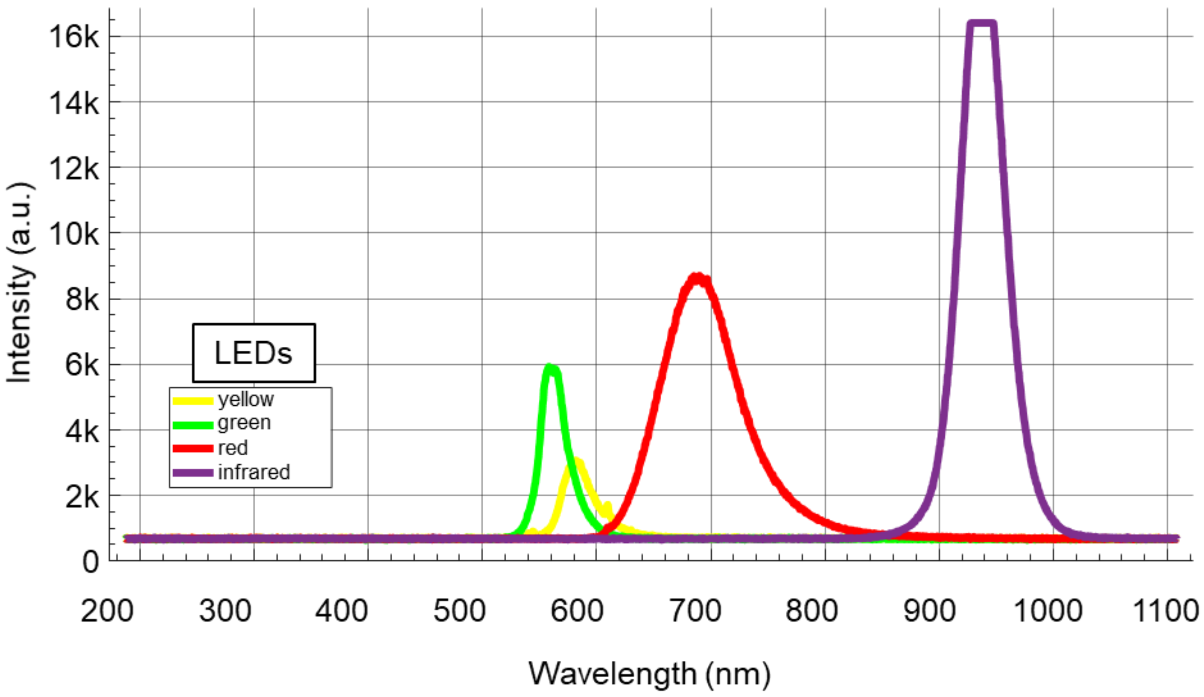

S21 Fig. Optical spectrometer test. Optical spectra taken by Ocean Optics HR2000+ of different LEDs controlled by PortaDrop.

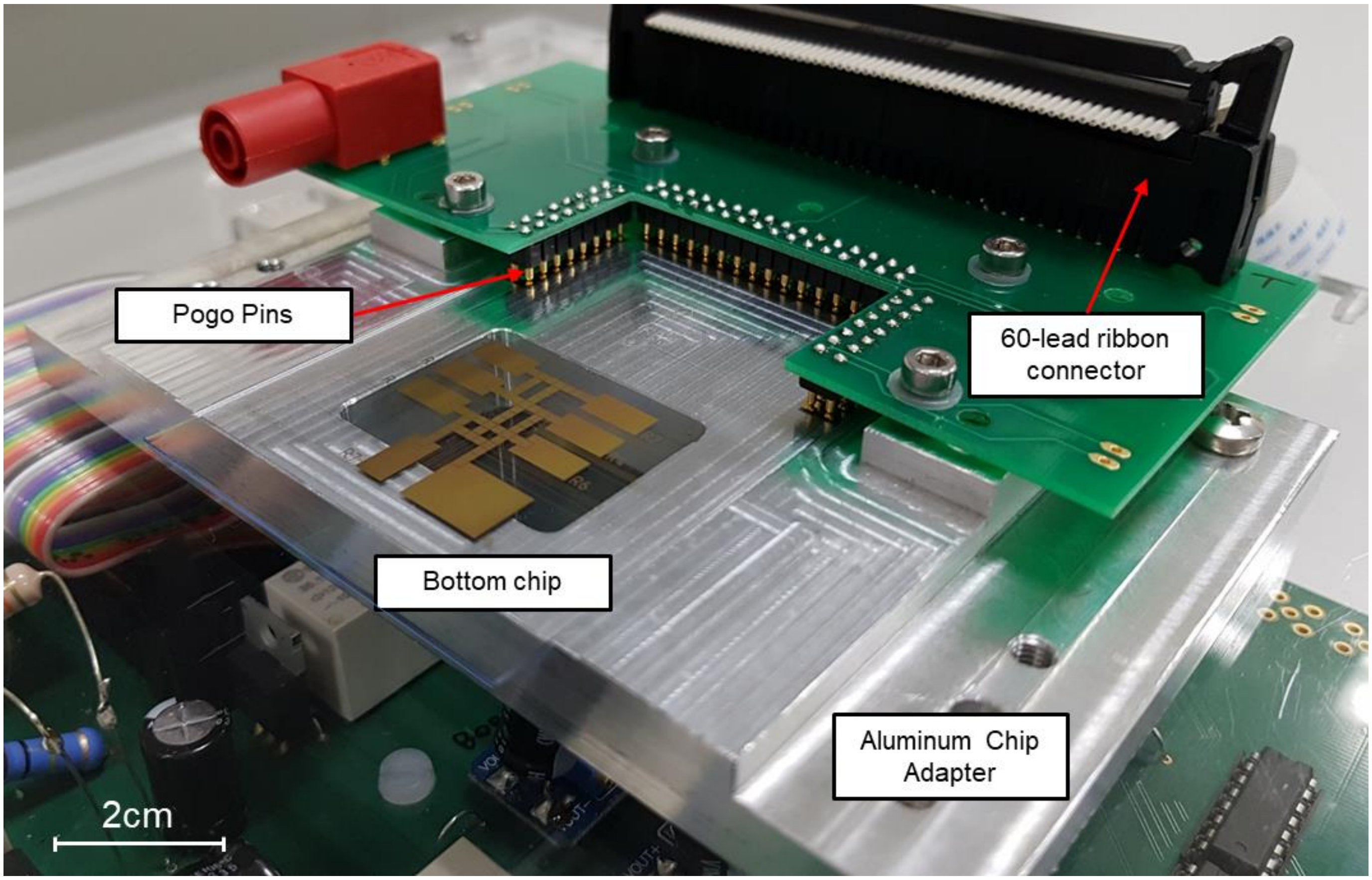

S22 Fig. Close-up of the bottom chip holder on top of the PortaDrop all-in-one box. The 60-lead ribbon cable is connected to the PCB and the bottom chip is contacted with pogo pins. The entire aluminum chip holder is screwed into the cover of the PortaDrop and can be application specifically replaced.

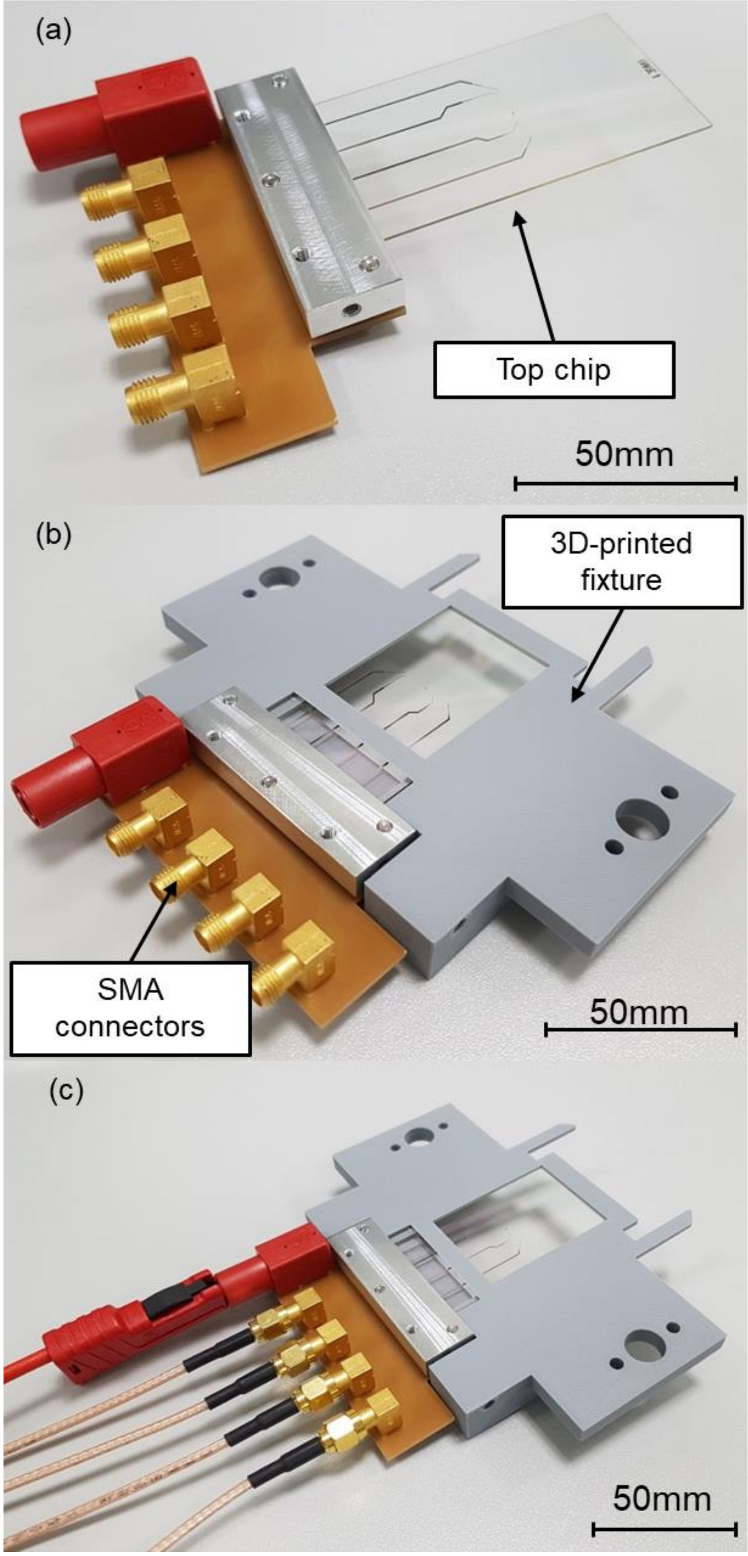

S23 Fig. 3D-printed top chip holder. Top chip holder made from (a) a PCB including pogo pins and connectors, (b) a 3D-printed fixture for easy alignment of bottom and top chip and (c) the ability to connect electrodes for electrochemical measurements included in the top chip as well as the ITO counter electrode for EWOD operation.

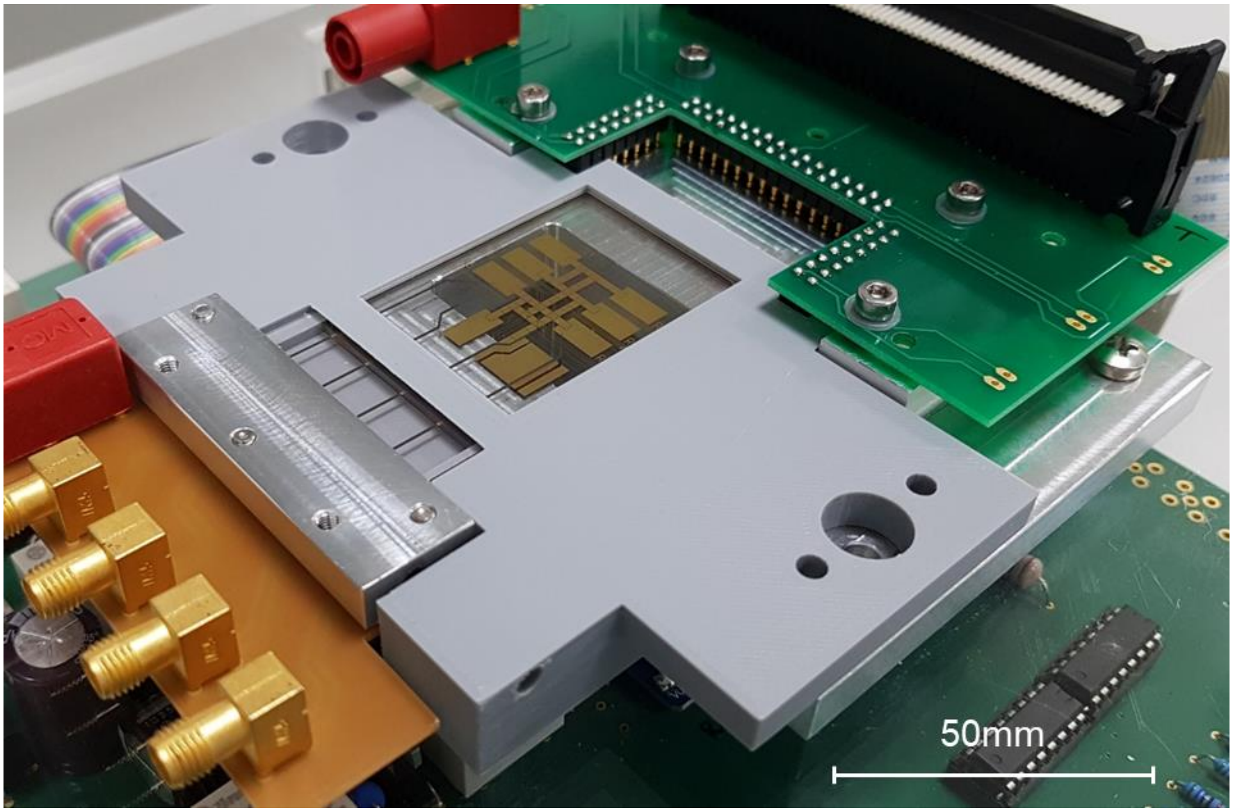

S24 Fig. Chip alignment. Assembly of bottom and top chip utilizing the 3D-printed fixture.

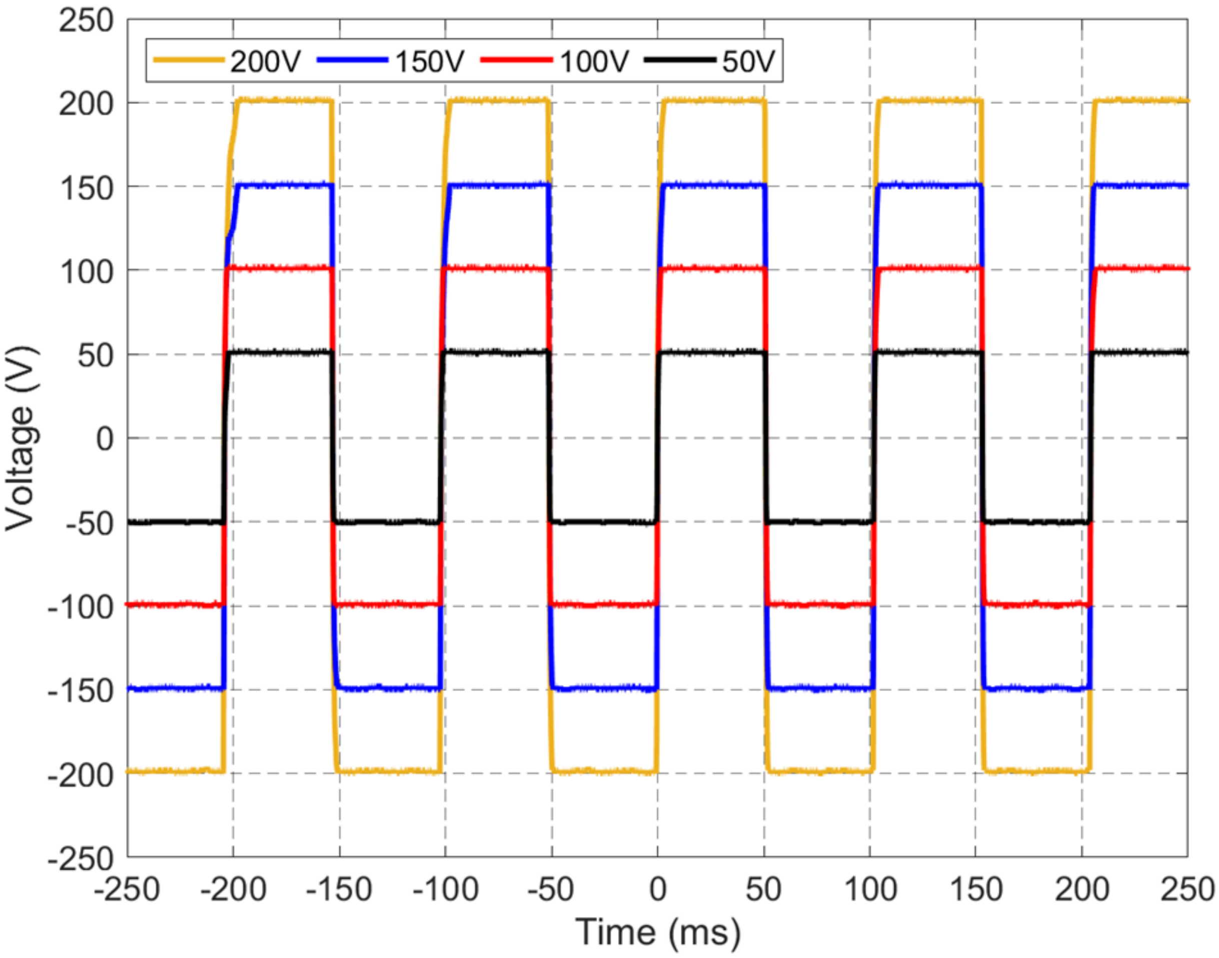

S25 Fig. H-bridge output characteristics. Output signal of the H-bridge for different voltages provided by an external high voltage source at a frequency of 10 Hz.

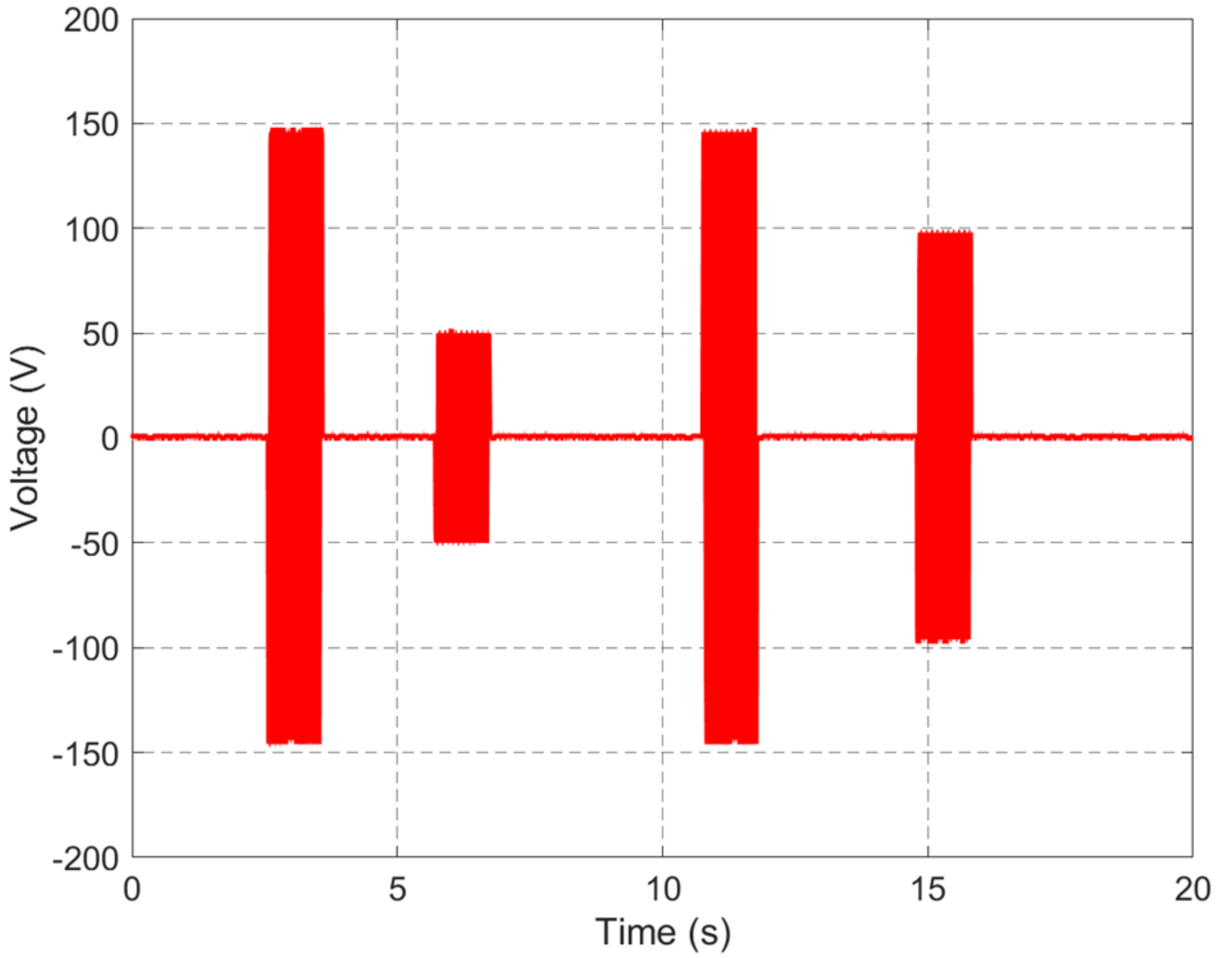

S26 Fig. Voltage profile of a specific channel. The voltage amplitude is varied and the frequency of the bipolar rectangle signal is adjusted to 100 Hz. The channel is switched on for 1 s and off when a stable voltage was confirmed by the voltage regulator.

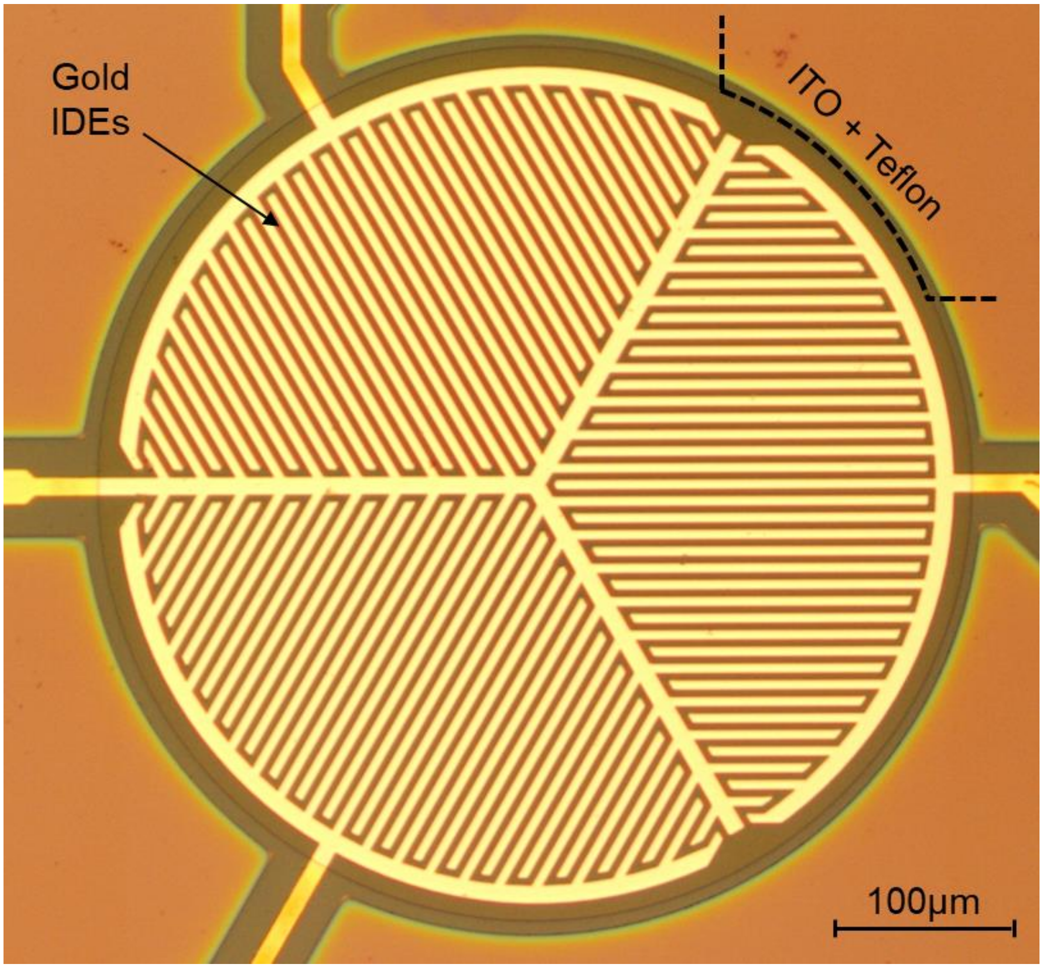

S27 Fig. Microscope picture of the gold IDEs. The outer area is covered with ITO and a hydrophobic Teflon layer, patterned circularly to allow access of the media to the sensor. The impedance between the star-shaped electrode and the three sector electrodes connected all together is measured.

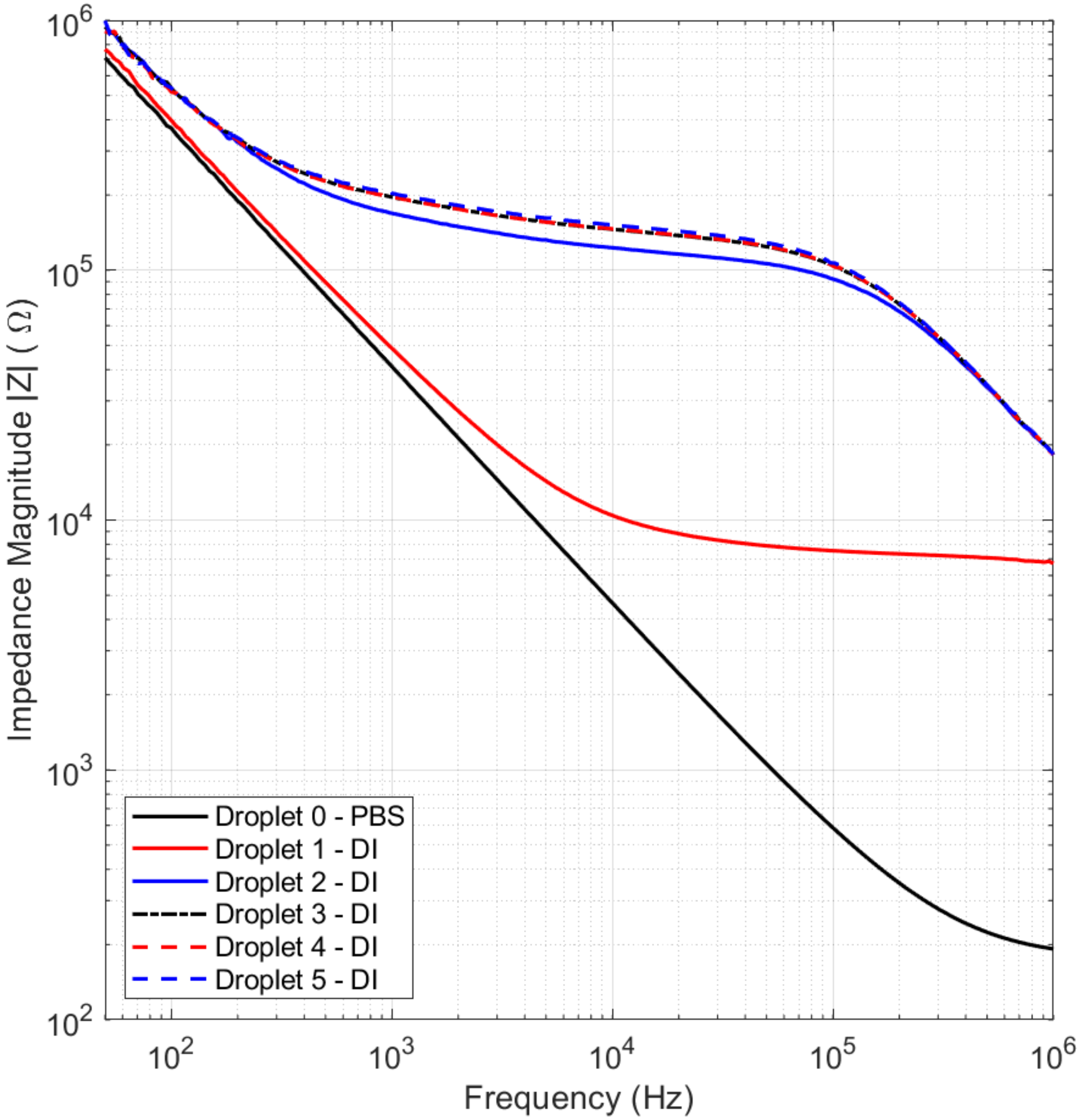

S28 Fig. Passive dispensing. Magnitude of the electrical impedance obtained in the virtual microwell during passive dispensing of a PBS droplet and five consequently dispensed DI water droplets

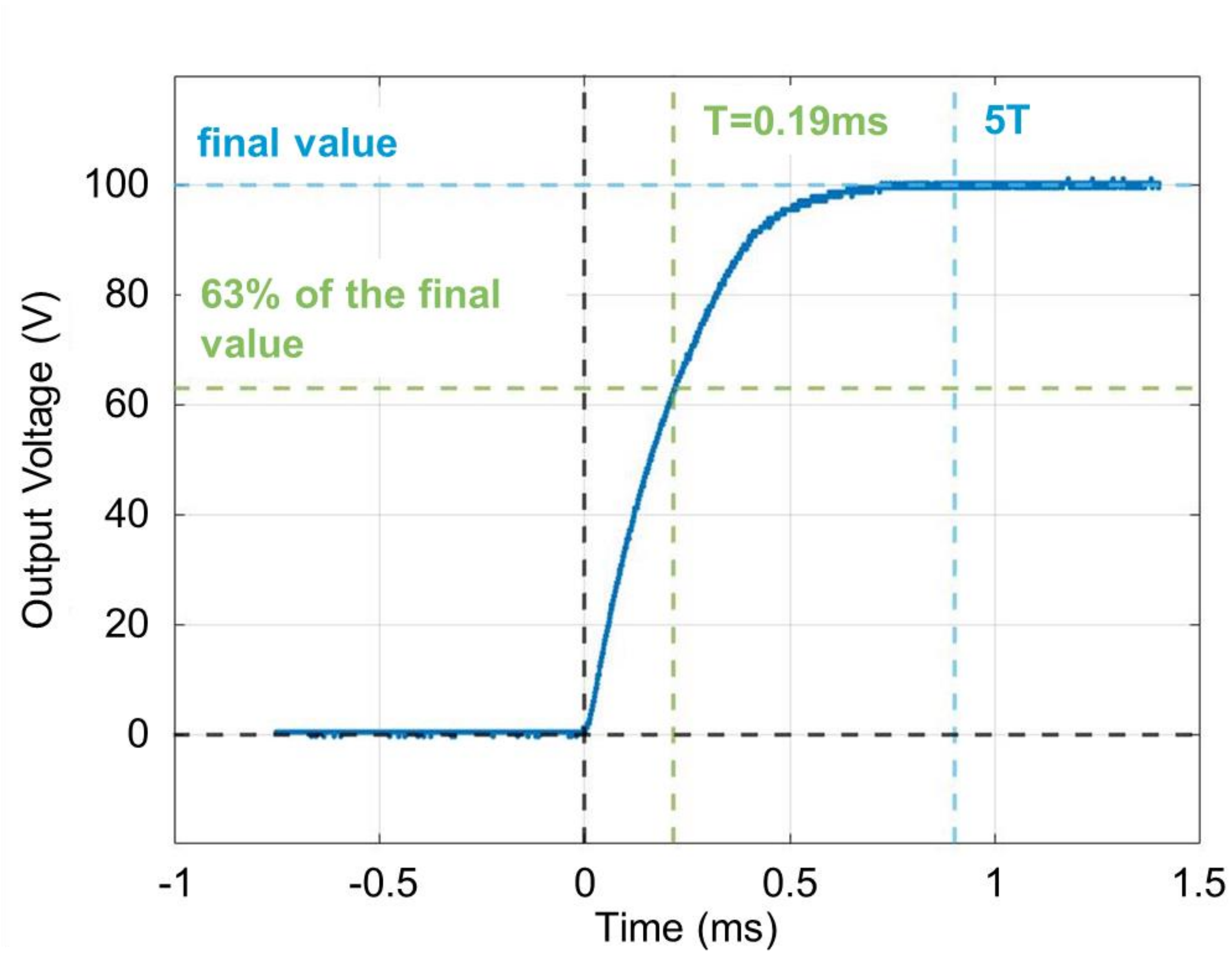

S29 Fig. Oscilloscope input. Time constant of the oscilloscope input in series with a 9MΩ divider resistor.

S1 Appendix. GUI Tabs and options.

Overview

- General: Overview of all connected devices. The internal microcontrollers are connected via I^2C and an arrow indicates proper communication. The implemented external devices change their state when connected. The refresh button updates the overview.
- Voltage Controller: Overview and control of the ATtiny45 microcontroller for voltage regulation. Data from the microcontroller can be requested using the refresh button. 'Set voltage' sends the set point for the voltage to the µC, which is then working in regulator mode controlling the voltage autonomous. 'Set Duty Cycle' allows to set the duty cycle of the PWM signal to the chosen value ***Important: The µC itself limits the voltage to stay in the specs of all circuit elements, when the voltage increases to undesired values.*** The button 'Set Frequency' adjusts the frequency of the PWM signal for the boost converter.
- Frequency Generator: Shows the actual frequency of the rectifier circuit. The value can be updated using the 'refresh' button. To change the frequency, a value for the new frequency can be entered and set using 'set Frequency'.
- Relay: Overview of all relay states on the mainboard. The relay position can be read via the ATmega32 on the mainboard and can be modified. ***Important: To avoid damage to e.g. external devices certain relays can't be set by the user. The software changes the state in a certain way and time pattern, when other relays are changed. The µC itself limits the voltage to stay in***

the specs of all circuit elements, when the voltage increases to undesired values.

- Video: Preview of the camera connected via CSI

Recipe

- Overview about all existing recipes and the actual recipe. The left column shows all stored recipes. This can for example be basic fluid operations (e.g. Dispense form reservoir R1). This recipe can be added to the actual recipe (right column) by clicking the '→' button, which can be a sequence of basic recipes or can be composed from individual task (see Add Task) or a combination of both. The 'duplicate' button extents the current recipe by the chosen step. The button 'remove' deletes certain steps, while 'clear' empties the entire recipe list. After composing an individual recipe, the recipe can be saved using the 'save' button, appears on the left column and can be used in future.

Project Data

- Measurements taken during the running recipe can be visualized. In addition, data of finished experiments are available
- Overview: Organization of the data which should be displayed. At first the 'project' needs to be selected and selected with the upper 'select' button. A list of all experiments carried out within the chosen project is generated below and the experiment can be chosen and selected. Below, a list of all recorded e.g. spectra (EIS or optical) is shown. Using the 'load' button the measurement is loaded to the corresponding tabs (see below).

- Spectrum: optical spectra taken with Ocean Optics HR2000+ USB spectrometer visualization. Example spectra of different LEDs (yellow, green, red and infrared) is shown in S20 Fig. of the supporting information
- Impedance: EIS spectra visualization
  - abs/phase: shows bode plots of the chosen measurements
  - nyquist: Nyquist plot representation of spectra
  - transient view: in case transient measurements have been taken during the experiment, the time dependent magnitude, phase, real part, imaginary part at one frequency is depicted.
- Add Task: In this section single recipe steps (tasks) can be added to the current recipe. Possible steps are:
  - Delay Task: Inserts a pause of specified duration
  - Frequency Task: Allows to change or to set the frequency within the recipe
  - Voltage Task: Adds a step for adjusting or turning on / off the voltage
  - Spectrometer Task: Adds an optical measurement to the recipe. The Ocean Optics HR2000+ USB spectrometer needs to be connected. A proper connection is checked at the start of the recipe.
  - Pad Task: Adds specified EWOD pads, which are turned on for a specified time to the recipe. Therefore, an overview of the path electrodes is depicted in the GUI. Multiple steps stringed together allow droplet movement over certain path electrodes. In addition, path electrodes can be activated manually to move droplets by hand.

- Imp Task: Adds an EIS measurement to the current recipe. Correct connection of the utilized instruments will be checked at the start of the recipe. The settings available are depicted in S16 Fig.

- Log: All status messages given by the software are recorded and shown here. The log file content during one experiment is also saved to the project folder.
- Preferences: Allows path settings to the project folder and the recipe folder.
- Manual: Developer functions for testing the semiconductor switching boards and connected GPIB devices easily.

S2 Appendix. Demo Recipe.

```
<! –– definition of one recipe –– >
<Recipe name="demo" comment=" ">
<! – define tasks within the recipe –– >
<! –– activate and configurate frequency generator –– >
<FreqTask name="f = 100 Hz" comment=" " freq="100"/>
<! –– activate / set voltage, mode fixed duty cycle (60%) –– >
<VoltageTask name="D = 60%" comment=" " mode="0" duty_cycle="0,6"/>
<! –– activate / set voltage, mode: regulator, set point 20 V) –– >
<VoltageTask name="V = 20V" comment=" " mode=" 1 " volt="20" wait_for_voltage="true"/>
<! – external voltage source –– >
<VoltageTask name="V = 20V" comment=" " mode="2" volt="20"/>
<! – Measurement of an optical spectrum –– >
<SpectrometerTask triggermode="0" integrationtime_us="1000" scansToAverage="1"/>
<! – Activation of defined EWOD pads (200ms) –– >
<PadTask name="power pads 2, 3, 15 for 200ms" comment=" " duration_ms="200">
<! – defintion of pads –– >
<Pad padNo="2"/>
<Pad padNo="3"/>
<Pad padNo="15"/>
</PadTask>
<! – Measurement of one EIS spectrum –– >
<ImpAnalyserTask>
<! – configuration of the device (external) in 4 wire setup –– >
<HP4294A gpibAddress="17" startFrequency="40" stopFrequency="11000010"
voltage="0,050000" points="201" bw="1" pointAverage="20" wireMode="4"/>
```

```
</ImpAnalyserTask>
<! – Measurement of one EIS spectrum –– >
<ImpAnalyserTask>
<! – configuration of the device (internal) in 2 wire setup –– >
<EmStatPico startFrequency="40" stopFrequency="200000" voltage="0,050000" points="400" pointAverage="9" wireMode="2"/>
</ImpAnalyserTask>
<! – transient measurement of EIS spectra – abort time (20s) –– >
<TransImpTask measurements_term_time="20">
<! –– configuration of the device (external) in 3 wire setup –– >
<Novocontrol gpibAddress="29" startFrequency="40" stopFrequency="11000010" voltage="0,050000" points="201" pointAverage="20" wireMode="3"/>
</TransImpTask>
<! – transient measurement of EIS spectra – abort number of spectra (15) –– >
<TransImpTask measurements_term_cnt="15">
<! –– configuration of the device (external) in 3 wire setup –– >
<Novocontrol gpibAddress="29" startFrequency="40" stopFrequency="11000010" voltage="0,050000" points="201" pointAverage="20" wireMode="3"/>
</TransImpTask>
<! – pause for 20 seconds –– >
<DelayTask delay_time_s="20">
</Recipe>
```
